# Designing Potent Anti-Cancer Agents: Synthesis and Molecular Docking Studies of Thieno[2,3-*d*][1,2,4]triazolo[1,5-*a*]pyrimidine Derivatives

**DOI:** 10.3390/molecules29051067

**Published:** 2024-02-29

**Authors:** Eman S. M. Elsenbawy, Zafer S. Alshehri, Nouf A. Babteen, Adel A.-H. Abdel-Rahman, Mai A. El-Manawaty, Eman S. Nossier, Reem K. Arafa, Nasser A. Hassan

**Affiliations:** 1 Department of Chemistry, Faculty of Science, Menofia University, Shbien El-Kom 32511, Egypt; eman_alsenbawy@yahoo.com (E.S.M.E.); adelnassar63@yahoo.com (A.A.-H.A.-R.); 2 Department of Medical Laboratories, College of Applied Medical Sciences, Shaqra University, Dawadmi 19257, Saudi Arabia; zaf@su.edu.sa; 3 Department of Biochemistry, College of Sciences, University of Jeddah, Jeddah 21577, Saudi Arabia; nababteen@uj.edu.sa; 4 Department of Pharmacognosy, Pharmaceutical Science Division, National Research Centre, Cairo 12622, Egypt; mayalyem@gmail.com; 5 Department of Pharmaceutical Medicinal Chemistry and Drug Design, Faculty of Pharmacy (Girls), Al-Azhar University, Cairo 11754, Egypt; dr.emannossier@gmail.com; 6 Drug Design and Discovery Laboratory, Biomedical Sciences Program, University of Science and Technology, Zewail City of Science and Technology, Ahmed Zewail Road, October Gardens, Cairo 12578, Egypt; rkhidr@zewailcity.edu.eg; 7 Synthetic Unit, Department of Photochemistry, Chemical Industries Research Institute, National Research Centre, Cairo 12622, Egypt

**Keywords:** synthesis, thieno[3,2-*d*]pyrimidin-4(3H)-one derivatives, thieno[2,3-*d*][1,2,4]triazolo[1,5-*a*]pyrimidines, anti-cancer agents, molecular docking, ADME

## Abstract

A new series of thieno[2,3-*d*][1,2,4]triazolo[1,5-*a*]pyrimidines was designed and synthesized using readily available starting materials, specifically, *β*-enaminoester. Their cytotoxicity was screened against three cancer cell lines, namely, MCF-7, HCT-116, and PC-3. 2-(4-bromophenyl)triazole **10b** and 2-(anthracen-9-yl)triazole **10e** afforded excellent potency against MCF-7 cell lines (IC_50_ = 19.4 ± 0.22 and 14.5 ± 0.30 μM, respectively) compared with doxorubicin (IC_50_ = 40.0 ± 3.9 μM). The latter derivatives **10b** and **10e** were further subjected to in silico ADME and docking simulation studies against EGFR and PI3K and could serve as ideal leads for additional modification in the field of anticancer research.

## 1. Introduction

Cancer is a global health crisis marked by increasing incidence and mortality rates. Prompt diagnosis and effective treatment strategies are crucial due to the growing number of individuals affected by cancer. The relentless pursuit of innovative therapeutic strategies for combating cancer has led to an increased emphasis on the design and synthesis of novel compounds with potent anti-cancer properties [1]. The thienopyrimidine scaffold has emerged as a frequently utilized chemical framework in drug development. Compounds containing thienopyrimidine exhibit structural and isoelectronic characteristics similar to purines, making them attractive in the production of pharmaceutical drugs [2,3] and have demonstrated significant pharmacological properties, including antibacterial [4,5,6], antiviral [7,8], anti-inflammatory [9,10], antiprotozoal [11], and anticancer activities [12,13,14,15].

The 1,2,4-triazole moiety is included in many anticancer medications on the market, such as vorozole, anastrozole and letrozole [16,17,18,19]; therefore, adding a 1,2,4-triazine moiety to the thienopyrimidine system is anticipated to significantly influence the cytotoxic activity [20]. Conversely, the combination of glycosides with heterocyclic molecules generated essential hybrids with biologically interesting properties, such as antiviral, anticancer, and antibacterial properties [21,22,23].

Since the epidermal growth factor receptor tyrosine kinase (EGFR) participates in the growth and progression of cancer, it represents a compelling target for cancer treatment [24]. EGFR is a desirable target in many illnesses such pancreatic cancer, breast cancer, and non-small cell lung cancer as well as those of the lung, ovarian, and breast regions [25,26,27,28]. Additionally, the signaling pathway that involves phosphatidylinositol 3-kinase (PI3K) is a key player in regulating cell viability, proliferation, migration, glucose metabolism, and death. It has been extensively investigated over the past few decades to create novel cancer therapies that target the earlier pathways [29,30].

**Figure 1:**
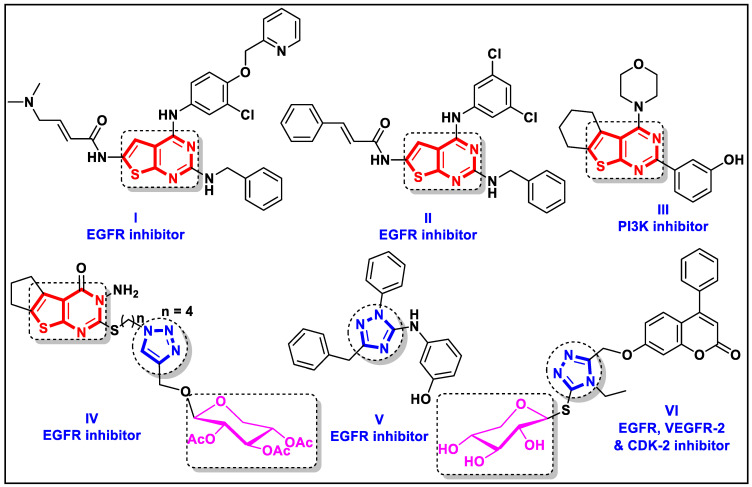
Various anticancer agents, including thieno[2,3-*d*]pyrimidine, triazole, and/or glycoside constituents with diverse mechanisms.

Several examples of diverse thienopyrimidine-containing drugs highlight their broad applications in various therapeutic areas. Pictilisib (GDC-0941) is currently under clinical investigation for its potential in addressing advanced solid tumors by inhibiting PI3K. Also, olmutinib has proven effective as an EGFR inhibitor, applied in the treatment of non-small cell lung cancer (NSCLC) [31]. Thieno[2,3-*d*]pyrimidine **I** was discovered to exceed the commercially available medicine lapatinib in terms of EGFR inhibition, which piqued a lot of interest [32]. Moreover, the derivative **II** illustrated powerful cytotoxicity against colorectal HCT-116, SW480, ovarian SKOV3, glioblastoma U87 and breast SKBR3 cancer cell lines, with IC_50_ values ranging from 3.83 to 11.94 μM when compared to erlotinib through EGFR inhibition behavior [33]. Thieno[2,3-*d*]pyrimidine **III** demonstrated significant antitumor efficacy via PI3K inhibition against NCI 60 cell lines [34]. Thieno[2,3-*d*]pyrimidine-1,2,3-triazole-glycoside **IV**, 1,2,4-triazole **V** and 1,2,4-triazole -glycoside **VI** also exhibit substantial anticancer activity, mainly against MCF-7, through their inhibitory activity against EGFR [35,36,37] (Figure 1).

Building on insights from the cited reports and our ongoing research in synthesizing biologically active compounds [22,38,39,40], this study focuses upon designing derivatives with the thieno[3,2-*d*]pyrimidin-4(3*H*)-one cores bearing 1,2,4-triazole and glycoside scaffolds. The effectiveness of these compounds will be assessed against MCF-7, HCT-116, and PC-3 cancer cell lines. The promising derivatives were further evaluated through molecular docking studies against EGFR and PI3K to predict their mechanism of action. Finally, in silico ADME studies were applied to determine the physicochemical and pharmacokinetic properties to facilitate valuable insights in development of more effective anticancer therapies.

## 2. Results and Discussion

### 2.1. Chemistry

**Scheme 1:**
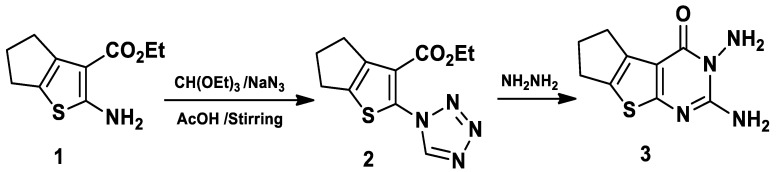
Synthesis of compound **3**.

**Scheme 2:**
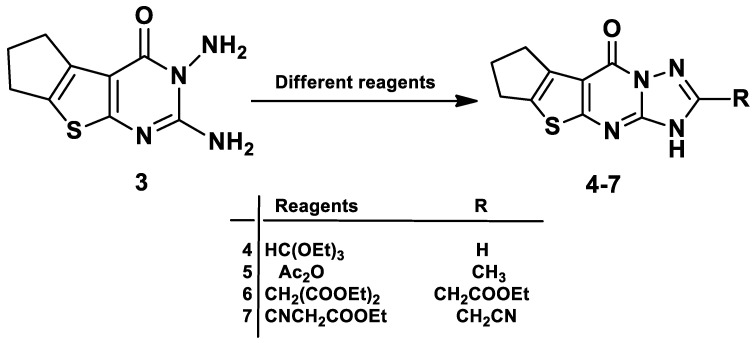
Synthesis of compounds **4**–**7**.

**Scheme 3:**
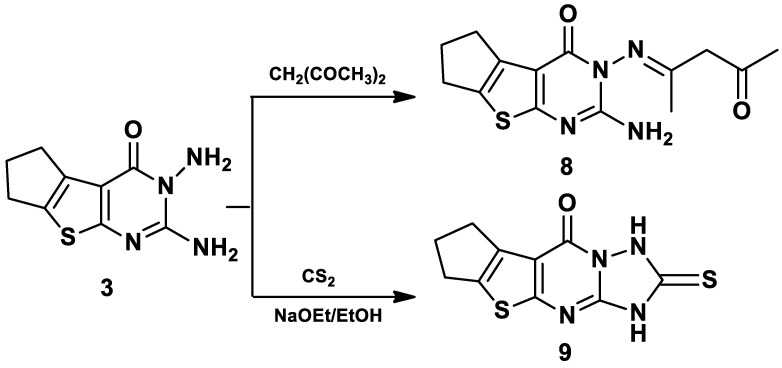
Synthesis of compounds **8**, **9**.

**Scheme 4:**
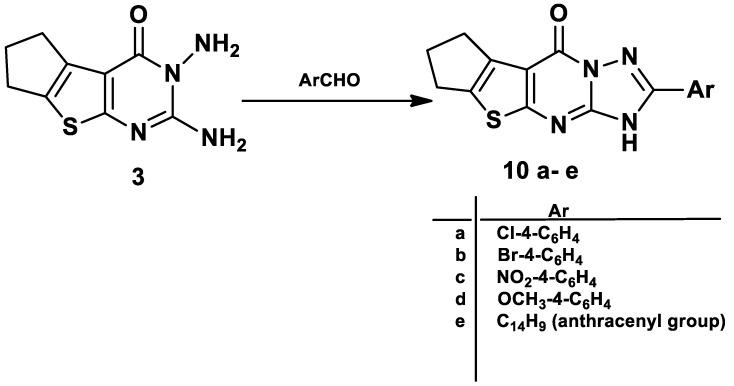
Synthesis of compounds **10a**–**e**.

**Scheme 5:**
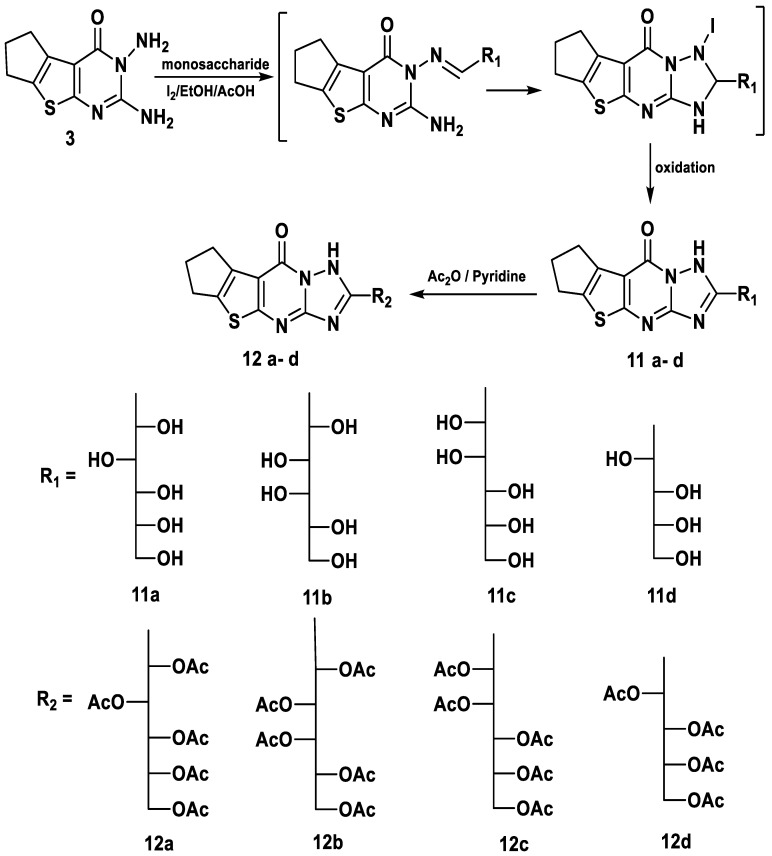
Synthesis of compounds **11a**–**d** and **12a**–**d**.

**Scheme 6:**
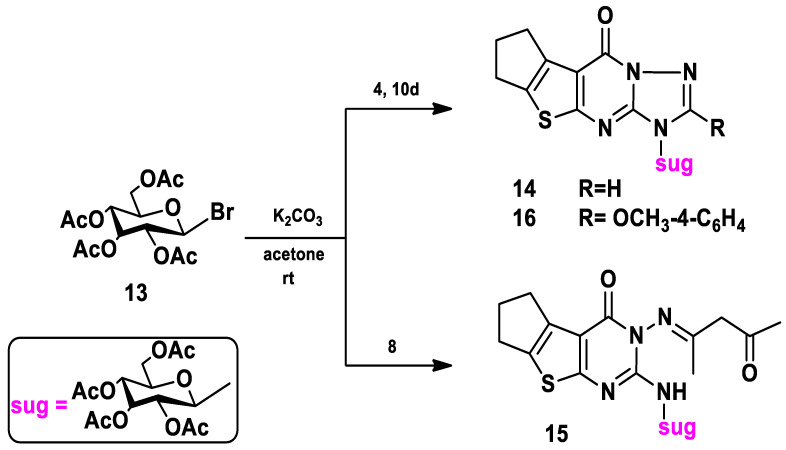
Synthesis of compounds **14**–**16**.

The synthetic approaches utilized for creating the intermediate and final compounds are illustrated in Scheme 1, Scheme 2, Scheme 3, Scheme 4, Scheme 5 and Scheme 6, respectively. In Scheme 1, ethyl 2-amino-5,6-dihydro-4H-cyclopenta[b]thiophene-3-carboxylate **1** underwent an efficient transformation to yield ethyl 2-(1H-tetrazol-1-yl)-5,6-dihydro-4H-cyclopenta[*b*]thiophene-3-carboxylate **2**. This conversion was achieved by subjecting compound **1** to a reaction with triethyl orthoformate and sodium azide in glacial acetic acid [41,42]. Subsequently, treatment of the resulting compound **2** with hydrazine hydrate led to the successful synthesis of cyclized thienotriazolopyrimidine **3**, obtained in a good yield. The chemical structure of compound **3** was confirmed through analysis of its analytical and spectral data (see experimental).

The reaction of 2,3-diamino-6,7-dihydro-3H-cyclopenta[4,5]thieno[2,3-*d*]pyrimidin-4(5H)-one **3** with triethyl orthoformate and acetic anhydride (Scheme 2) readily provided the cyclized thienotriazolopyrimidins **4** and **5**, respectively. The confirmation of the chemical structures of compounds **4** and **5** was achieved through the analysis of both analytical and spectral data. For instance, in the case of compound **4**, the IR spectrum revealed absorption bands at 3309 and 1670 cm^−1^, corresponding to the NH and C=O groups, respectively. Additionally, in the ^1^H NMR spectrum, a singlet signal at 7.54 ppm was observed, which was attributed to the triazolo proton. Moreover, the ^13^C NMR spectrum displayed distinct signals at 132.19 and 166.61 ppm, corresponding to the triazole CH and CO, respectively.

Upon heating compound **3** with diethylmalonate, the ethyl acetate ester **6** was formed. The IR spectrum of compound **6** displayed prominent absorption bands at 3395, 1718 and 1684 cm^−1^ due to NH_2_, ester, and amidic C=O groups. The ^1^H NMR spectrum revealed expected triplet and quartet signals corresponding to the ester group, in addition to a singlet at 3.33 ppm, corresponding to the CH_2_CO protons.

The synthesis of the 3-cyanomethyl derivative **7** was accomplished with a satisfactory yield by subjecting compound **3** to a reaction with ethyl cyanoacetate at 180 °C. In the ^1^H NMR spectrum, a singlet appeared at 4.32 ppm, which was ascribed to the CH_2_ protons. Additionally, the IR spectrum displayed distinctive signal of the nitrile group at 2265 cm^−1^ (Scheme 2).

Subjecting compound **3** to heat in the presence of excess acetyl acetone resulted in the formation of the 1-methyl-3-oxobutylideneamino derivative **8** (Scheme 3). In its ^1^H-NMR spectrum, the upfield resonance of the N=C-CH_3_ protons and the methyl carbon provided confirmation of an E configuration at C1 [43,44].

By subjecting compound **3** to heat in the presence of carbon disulfide in ethanol with sodium ethoxide, we successfully synthesized the 2-thioxo analog **9** (Scheme 3). The validation of its structure was established through multiple analyses, including the mass spectrum, where a molecular ion peak at *m*/*z* 262 (35.1%) was observed. Additionally, in the 1H NMR spectrum, two singlets were detected at δ 10.57 and 10.90 ppm, which were attributed to the 2NH protons and found to be exchangeable with D_2_O.

The fusion of compound **3** with different aromatic aldehydes, namely 4-chlorobenzaldehyde, 4-bromobenzaldehyde, 4-nitrobenzaldehyde, 4-methoxybenzaldehyde, and anthracenaldehyde, in an oil bath at 180 °C led to the formation of the desired thienotriazolopyrimidines **10a**–**e** (Scheme 4). The compounds **10a**–**e** displayed strong IR absorption bands at 3396–3308 cm^−1^ and 1680–1658 cm^−1^ indicating the presence of NH and C=O groups, respectively. In the analysis of the ^1^H NMR spectra of compounds **10a**–**e**, the absence of a singlet corresponding to the N=CH proton, expected for the azomethine proton, confirmed the formation of the cyclized product. 

The oxidative condensation of monosaccharides, namely, (D)-glucose, (D)-galactose, (D)-mannose, and (D)-arabinose with **3**, occurred readily at room temperature using a catalytic amount of iodine in acetic acid. The reaction was completed within 6–12 h, as monitored by thin-layer chromatography, leading to the formation of **11a**–**d**, which, upon acylation, resulted in the formation of the acylated products **12a**–**d**. The structures of the new deacylated and acylated products were established based on their microanalytical and spectroscopic data (see experimental and Scheme 5).

The coupling of compounds **4**, **8,** and **10d** with 2,3,4,6-tetra-O-acetyl-α-D-glucopyranosyl bromide **13** in acetone and potassium carbonate afforded the *N*-glycosylated nucleosides **14**, **15,** and **16** in good yields (73, 62, and 65%, respectively (Scheme 6). Thin layer chromatography (chloroform/methanol = 10:1) indicated formation of the pure compounds. The structures of the products **14**, **15,** and **16** were confirmed by elemental analyses and spectral data (IR, ^1^H NMR, ^13^C NMR) (see Experimental). For instance, analytical data for compound **16** revealed a molecular formula of C_31_H_32_N_4_O_11_S (M^+^ 668.67). The ^1^H NMR spectrum showed the anomeric proton of the glucose moiety as a doublet at δ 5.03–5.25 ppm with a coupling constant *J* = 10.5 Hz indicating *β*-configuration of the anomeric center. The other protons of the glucopyranose ring resonated at δ 3.84–6.52 ppm, while the four acetoxy groups appeared as four singlets at 1.13–2.21 ppm.

### 2.2. Cytotoxicity Evaluation

**Figure 2:**
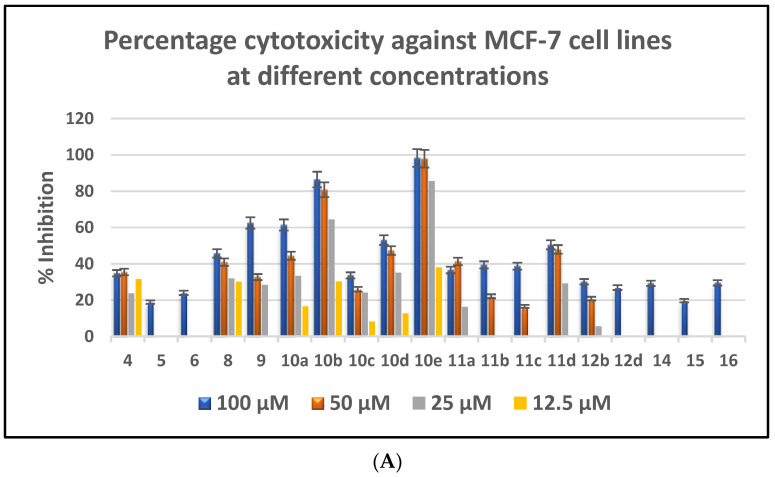

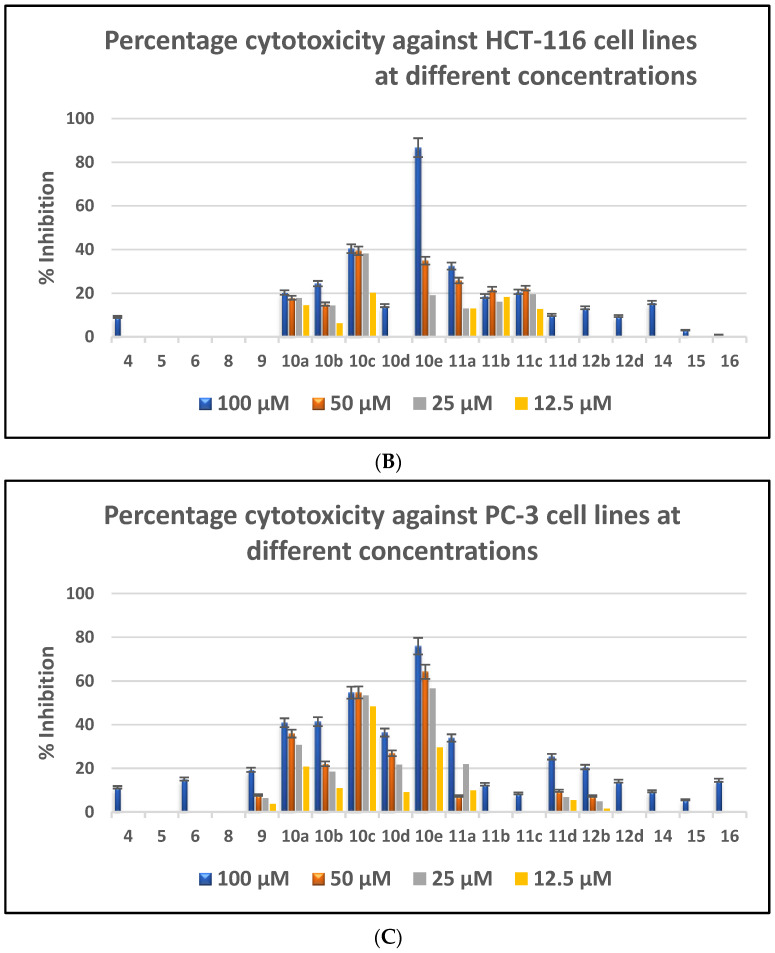
(**A**–**C**) illustrate preliminary cytotoxic activities of cyclopenta[4,5]thieno[2,3-*d*][1,2,4]triazolo [1,5-a]pyrimidin-9(6H)-ones **4**–**16** according to the MTT assay against human cancer MCF-7, HCT-116, and PC-3 cell lines, respectively at different concentrations in µM.

Using human breast MCF-7, colorectal HCT-116, and prostate PC-3 cancer cell lines, preliminary antiproliferative effectiveness of cyclopenta[4,5]thieno[2,3-*d*][1,2,4]triazolo[1,5-*a*]pyrimidin-9(6*H*)-ones **4**–**16** was established in vitro through MTT assay [45,46,47] at different concentrations (100, 50, 25 and 12.5 μM) (Figure 2). The promising derivatives that displayed cytotoxic potency ≥60% at a concentration of 100 μM were subjected for calculation of their IC_50_ as indicated in Table 1 comparing their cytotoxicity with doxorubicin as a reference drug. It was noted that the derivatives **9**, **10a**, **10b,** and **10e** were more effective on MCF-7 cell lines than other screened cell lines (IC_50_ range 14.5–17.8 μM against MCF-7). Moreover, the analogue **10e** displayed broad spectrum inhibitory activity against all examined cell lines (IC_50_ = 14.5, 57.01 and 25.23 μM against MCF-7, HCT-116, and PC-3, respectively).

By inspection of the previous results, it was observed that 2-thioxotriazole **9** and 2-(4-chlorophenyl)triazole **10a** afforded moderate cytotoxicity against MCF-7 cell lines in comparison with doxorubicin (IC_50_ = 71.8 ± 1.05, 60.6 ± 0.45, and 40.0 ± 3.9 μM, respectively). However, 2-(4-bromophenyl)triazole **10b** and 2-(anthracen-9-yl)triazole **10e** revealed excellent and superior cytotoxic activity with respect to the standard (IC_50_ = 19.4 ± 0.22 and 14.5 ± 0.30 μM, respectively).

Additionally, the cytotoxic activity of the highly effective derivatives **10b** and **10e** against the human skin normal cell line (BJ-1) was investigated using the MTT assay (Table 1) in order to identify their safety profiles. The IC_50_ values of the latter derivatives against normal cells were 221.7 ± 30, 34.81 ± 4.5, and 49.25 ± 1.08 µM, respectively. Thus, while **10e** showed comparable activity to doxorubicin, **10b** was far less cytotoxic to BJ-1. It can therefore be concluded that **10b** presents a safer cytotoxicity profile than **10e**.

In summary, the SAR (structure–activity relationship) analysis of this series of thieno[3,2-d]pyrimidin-4(3H)-one derivatives revealed that the presence of the cyclopenta[4,5]thieno[2,3-*d*][1,2,4]triazolo[1,5-*a*]pyrimidinone core enhanced the cytotoxicity of the tested compounds against cancer cell lines, with selectivity favoring MCF-7 cancer cell lines. For instance, derivative 8 exhibited low cytotoxicity. On the other hand, the nature of the substituent at the 2-position significantly influenced the anti-proliferative activity against MCF-7, with better results for derivatives bearing aryl moieties containing larger and lipophilic functional groups, in the order of **10e** > **10b** > **10a** > **9** (anthracenyl > 4-bromophenyl > 4-chlorophenyl > mercapto groups, respectively). Derivatives with hydrophilic and/or electron-donating functionalities, such as **10c** and **10d** (4-nitrophenyl and 4-methoxyphenyl analogs, respectively), were less potent in this regard. Conversely, derivatives with aliphatic substituents at the 2-position did not exhibit potent cytotoxicity (derivatives **4**–**7**). Additionally, it was observed that the introduction of sugar moieties in derivatives **11a**–**d**, **12a**–**d**, and **14**–**16** did not significantly contribute to the cytotoxicity of this compound series. Finally, derivative **10d**, with the 2-anthracenyl group, displayed a broad-spectrum anticancer activity as the only cytotoxic derivative against the three cancer cell lines: MCF-7, HCT-116, and PC-3.

Investigating the targets’ absorption, distribution, metabolism, and excretion (ADME) is a critical first step in selecting the best possible medication candidate. Swiss-ADME, a free online tool, made this anticipated examination easier [48,49,50]. Veber’s (molecule with number of rotatable bonds ≤ 10, TPSA ≤ 140 Å^2^) and Lipinski’s (MW ≤ 500, MLogP ≤ 4.15, number of hydrogen bond acceptors ≤ 10 and number of hydrogen bond donors ≤ 5) rules should be taken into consideration while selecting an oral drug. Anthracenyl derivative **10e** had one violation with MLogP > 4.15, whereas 4-bromophenyl **10b** seemed to be in accordance with the prior rules with no violations (Table 2).

**Figure 3:**
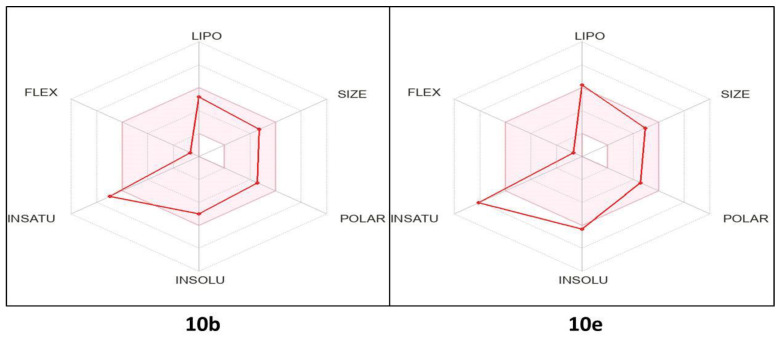
The bioavailability radar chart of cyclopenta[4,5]thieno[2,3-*d*][1,2,4]triazolo[1,5-*a*]pyrimidinones **10b** and **10e**. The predicted values for the targets **10b** and **10e** were identified as red lines, and the optimum range for each oral bioavailability parameter was presented in the pink area.

The cyclopenta[4,5]thieno[2,3-*d*][1,2,4]triazolo[1,5-*a*]pyrimidinone **10b** was identified to be in the optimal range (pink zone) with respect to the critical variables lipophilicity, polarity, size, solubility and flexibility, as illustrated in Figure 3 of the bioavailability radar chart; however, the derivative **10e** departed away from solubility and saturation. 

**Figure 4:**
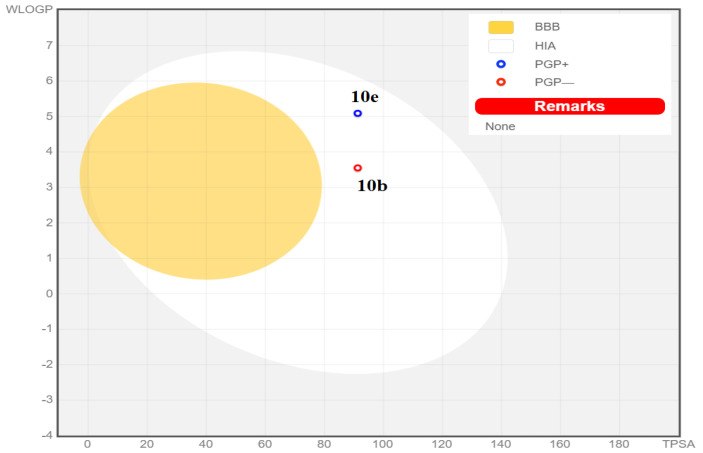
Boiled egg diagram revealing the characteristics of cyclopenta[4,5]thieno[2,3-*d*][1,2,4]triazolo[1,5-*a*]pyrimidin-9(6*H*)-ones **10b** and **10e** regarding the ability to penetrate the blood–brain barrier; be absorbed by the gastrointestinal tract; to be PGP+: a substrate of p-glycoprotein or PGP-: a non-substrate of p-glycoprotein.

Figure 3 and Table 3 represent the investigated pharmacokinetic characteristics of the promising cyclopenta[4,5]thieno[2,3-*d*][1,2,4]triazolo[1,5-*a*]pyrimidinones **10b** and 10e. Both derivatives **10b** and **10e** were anticipated to have a high gastrointestinal absorption with no brain penetration (within the white region and away from the yellow one of the boiled egg chart). The red dot of **10b** in Figure 4 suggests that it has a restricted ability to efflux out of the cell, representing its maximal potency as it’s not a substrate for p-glycoprotein (P-gp, drug efflux transporter), unlike compound **10e** (blue dot, P-gp substrate). These substances also have a satisfactory bioavailability value of 0.55 and no pain alert.

Based upon the excellent outcomes retrieved from the cytotoxic evaluation of both cyclopenta[4,5]thieno[2,3-*d*][1,2,4]triazolo[1,5-*a*]pyrimidin-9(6*H*)-ones **10b** and **10e** against MCF-7, HCT-116, and PC-3 cell lines and their promising drug-like characteristics, a docking simulation was carried out to predict their mechanism of action. The docking procedure was carried out using MOE-Dock (Molecular Operating Environment) software version 2014.01 [51]. First, the process was validated by re-docking the native ligands, erlotinib and quinolone LXX, within the active sites of EGFR and PI3K (PDB codes: 1M17 and 3L54, respectively) [52,53], providing energy score values of −11.75 and −10.60 kcal/mol with relatively minor values of RMSD (1.22 and 0.78 Å, respectively), between the co-crystallized ligands and their docked positions.

**Figure 5:**
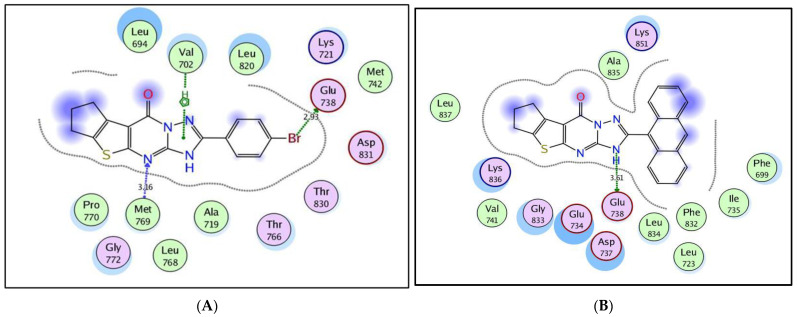
(**A**,**B**) views illustrate the 2D binding features of cyclopenta[4,5]thieno[2,3-*d*][1,2,4]triazolo[1,5-*a*]pyrimidin-9(6*H*)-ones **10b** and **10e** within the active site of EGFR kinase (PDB code: 1M17), respectively.

As depicted in Figure 5, the screened cyclopenta[4,5]thieno[2,3-*d*][1,2,4]triazolo[1,5-*a*]pyrimidin-9(6*H*)-ones **10b** and **10e** revealed promising binding within the active site of EGFR with observed energy score values of −11.46 and −9.33 kcal/mol compared with the original ligand, erlotinib. Regarding **10b**, the pyrimidine-N4 acted as a H-bond acceptor for the backbone NH of Met769 (distance: 3.16 Å), while the triazole core showed arene-H interaction with Val702. Additional H-bond donor interaction was established between the Br atom and the sidechain of Glu738 (distance: 2.93 Å). Upon replacement of the 4-bromophenyl with anthracen-9-yl moiety in **10e**, the molecule pushed away, thus losing the binding with key amino acid Met769 and facilitating H-bonding with the sidechain of Glu738 through the triazole-NH (distance: 3.61 Å).

**Figure 6:**
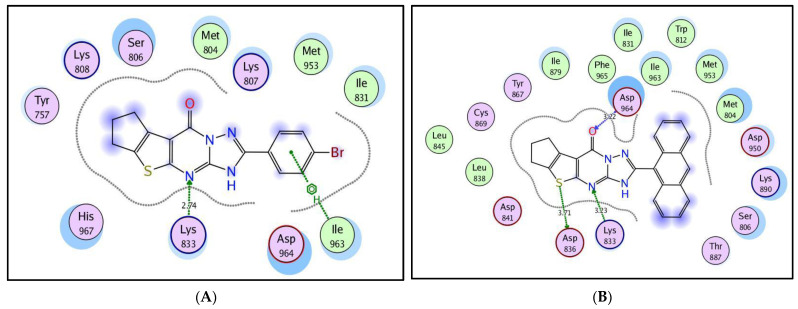
(**A**,**B**) views illustrate the 2D binding features of cyclopenta[4,5]thieno[2,3-*d*][1,2,4]triazolo[1,5-*a*]pyrimidin-9(6*H*)-ones **10b** and **10e** within the active site of PI3K (PDB code: 3L54), respectively.

On the other hand, with regard to interaction with PI3K, both derivatives **10b** and **10e** acted as H-bond acceptors with the key amino acids Lys833, according to Figure 6. Additionally, the centroid of the 4-bromophenyl afforded arene-H interaction with Ile963 in **10b**. However, the anthracen-9-yl analogue **10e** was fitted better as the sulfur and oxygen atoms of cyclopenta[4,5]thieno[2,3-*d*][1,2,4]triazolo[1,5-*a*]pyrimidinone core established several H-bonds with the amino acid Asp836 and Asp964 (distance: 3.71 and 3.22 Å, respectively).

The presence of cyclopenta[4,5]thieno[2,3-*d*][1,2,4]triazolo[1,5-*a*]pyrimidin-9(6*H*)-one core facilitated fixation within EGFR and PI3K active sites. Binding and selectivity with EGFR were improved by substituting a less bulky moiety at position-2 in **10e**, whereas a bulkier moiety shifted selectivity towards PI3k in **10e**.

## 3. Materials and Methods 

### 3.1. General Information

All melting points are uncorrected, were measured by using an electro-thermal IA 9100 apparatus (Shimadzu, Kyoto, Japan). Microanalyses were carried out at the Microanalytical center (Faculty of Science, Cairo University, Egypt). IR spectra were carried out on JASCO FT/IR 6100 Japan spectrometer (National Research Centre, Cairo, Egypt) using KBr discs. ^1^H-NMR spectra were measured in DMSO using JEOL EX-270 run for ^1^H-NMR at 270 MHz; JEOL ECA-500 run for ^1^H-NMR at 500 MHz and run for ^13^C-NMR at 125 MHz spectrometers. Chemical shifts were expressed in parts per million (δ ppm) against tetramethylsilane (TMS) as an internal standard. The coupling constant *J* is expressed in Hz. Mass spectra were recorded on GCMS Finnigan mat SSQ 7000 spectrometer. UV–Vis was recorded using (Shimadzu spectrophotometer). TEM was recorded by using High-Resolution Transmission Electron Microscopy (HRTEM) JEOL (JEM-2100 TEM). All reactions were followed up by thin layer chromatography (TLC). Aluminum sheets were used recoated with UV fluorescent silica gel (Merck Kieselgel 60 F_245_). It was visualized using a UV lamp and iodine vapor. Fine chemicals were of analytical grade; Selenious acid (H_2_SeO_3_) (Aldrich, Burlington, MA, USA), ascorbic acid (99%, Aldrich). All solvents were dried before being used. Refer to Supplementary Materials for 1H-NMR and 13C-NMR spectra of sample compounds.

### 3.2. Chemistry

*Ethyl 2-amino-5,6-dihydro-4H-cyclopenta[b]thiophene-3-carboxylate (* **1** *)*

Sulfur (3.16 g, 99 mmol) was added over a period of 15 min to a mixture of cyclopentanone (7.96 mL, 90 mmol), ethyl cyanoacetate (10.55 mL, 99 mmol) and diethylamine (10 mL, 99 mmol) in absolute ethanol that was stirred under reflux at 50 °C for 2 h. The reaction mixture was cooled, precipitated solid was filtered off, dried well, and recrystallized from ethanol to afford in 87% yield as yellow crystal.

*Ethyl 2-(1H-tetrazol-1-yl)-5,6-dihydro-4H-cyclopenta[b]thiophene-3-carboxylate (* **2** *)*

A suspension of **1** (9.95 g, 50 mmol), triethyl orthoformate (38.29 mL, 230 mmol), and sodium azide (3.90 g, 60 mmol) in glacial acetic acid (40 mL) was stirred under reflux for 2 h. The reaction mixture was cooled to room temperature and 7 mL of conc. HCl was added. The separated solid was filtered off and the filtrate was evaporated under reduced pressure. The remaining residue was recrystallized from ethanol to afford brown crystal in 65% yield.

*2,3-Diamino-6,7-dihydro-3H-cyclopenta[4,5]thieno[2,3-d]pyrimidin-4(5H)-one (* **3** *)*

A suspension of **2** (2.64 g, 10 mmol) in hydrazine hydrate (15 mL) was heated under reflux for 7 h, then cooled and diluted well with 50 mL water. The precipitated solid was filtered off, washed with diethylether, dried well, and recrystallized from ethanol. Yield: 80%; Mp: 285–287 °C. IR (KBr, cm^−1^): 3411, 3367 (2NH_2_), 2924 (CH), 1660 (C=O). ^1^H NMR (DMSO-*d_6_*): *δ* 2.36–2.43 (m, 2H, CH_2_), 2.80–2.84 (m, 2H, CH_2_), 5.85, 7.09 (2s, 4H, 2NH_2_). MS (*m*/*z*, %): 222 (M^+^, 60.1), 207 (100.0), 178 (91.7), 151 (17.2), 88 (13.7). Anal. Calcd for C_9_H_10_N_4_OS (222.27): C, 48.63; H, 4.53; N, 25.21; S, 14.43. Found: C, 4 8.42; H, 4.35; N, 25.11; S, 14.41.

*7,8-Dihydro-3H-cyclopenta[4,5]thieno[2,3-d][1,2,4]triazolo[1,5-a]pyrimidin-9(6H)-one (* **4** *)*

A suspension of **3** (0.44 g, 2.0 mmol) in triethlorthformate (2.96 g, 20 mmol) was heated under reflux for 4 h. The reaction mixture was left to cool, and the precipitated solid was filtered off, washed with cold ethanol, dried well, and recrystallized from dimethyl formamide. Yield: 78%; Mp: >300 °C. IR (KBr, cm^−1^): 3309 (NH), 3043 (CH-Aromatic), 1670 (C=O), 1618 (C=N). ^1^H NMR (DMSO-*d_6_*): *δ* 2.31–2.38 (m, 2H, CH_2_), 2.79–2.88 (m, 4H, CH_2_), 7.54 (s, 1H, triazole-CH), 11.25 (s, 1H, NH). ^13^C NMR (DMSO-*d*_6_): *δ* 27.60 (cyclopentane C-3), 27.81 (cyclopentane C-4), 29.62 (cyclopentane C-5), 119.47 (pyrimidine C-5), 132.19 (triazole C-2), 139.05 (thiophene C-4), 139.66 (thiophene C-5), 147.51 (triazole C-5), 157.20 (pyrimidine C-6), 166.61 (C=O). Anal. Calcd for C_10_H_8_N_4_OS (232.26): C, 51.71; H, 3.47; N, 24.12; S, 13.81. Found: C, 51.87; H, 3.34; N, 24.03; S, 13.83.

*2-Methyl-7,8-dihydro-3H-cyclopenta[4,5]thieno[2,3-d][1,2,4]triazolo[1,5-a]pyrimidin-9(6H)-one (* **5** *)*

A mixture of **3** (0.44 g, 2.0 mmol) in acetic anhydride (5 mL) was heated under reflux for 36 h. The reaction mixture was left to cool, and the precipitated solid was filtered off, washed with ethanol, dried well, and recrystallized from dimethyl formamide/ethanol (1:2). Yield: 82%; Mp: 93–95 °C. IR (KBr, cm^−1^): 3363 (NH), 2988 (CH), 1672 (CO), 1624 (CN), ^1^H NMR (DMSO-*d_6_*): *δ* 2.24 (s, 3H, CH_3_), 2.35–2.40 (m, 2H, CH_2_), 2.80–2.88 (m, 4H, CH_2_), 10.06 (s, 1H, NH). MS (*m*/*z*, %): 246 (M^+^, 75.5), 86 (62.4), 84 (100.0), 51 (38.8). Anal. Calcd for C_11_H_10_N_4_OS (246.29): C, 53.64; H, 4.09; N, 22.75; S, 13.02. Found: C, 53.35; H, 3.91; N, 22.69; S, 13.00.

*Ethyl 2-(9-oxo-6,7,8,9-tetrahydro-3H-cyclopenta[4,5]thieno[2,3-d][1,2,4]triazolo[1,5-a]-pyrimidin-2-yl)acetate (* **6** *)*

A suspension of **3** (0.44 g, 2.0 mmol) in diethylmalonate (3.27 g, 20 mmol) was heated under reflux for 4 h. The reaction mixture was left to cool, and the precipitated solid was filtered off, washed with cold ethanol, dried well, and recrystallized from dimethylformamide. Yield: 82%; Mp: >300 °C. IR (KBr, cm^−1^): 3395 (NH), 2971 (CH), 1718, 1684 (2C=O), 1610 (C=N). ^1^H NMR (DMSO-*d*_6_): *δ* 1.35–1.38 (t, *J = 7.2 Hz*, 3H, OCH_2_CH_3_), 2.37–2.43 (m, 2H, CH_2_), 2.83–2.95 (m, 4H, CH_2_), 3.33 (s, 2H, CH_2_CO), 4.32–4.37 (q, *J = 7.1 Hz*, 2H, OCH_2_CH_3_), 10.30 (s, 1H, NH). Anal. Calcd for C_14_H_14_N_4_O_3_S (318.35): C, 52.82; H, 4.43; N, 17.60; S, 10.07. Found: C, 52.62; H, 4.66; N, 17.55; S, 10.03.

*2-(9-Oxo-6,7,8,9-tetrahydro-3H-cyclopenta[4,5]thieno[2,3-d][1,2,4]triazolo[1,5-a]pyrimidin-2-yl)acetonitrile (* **7** *)*

A mixture of **3** (0.44 g, 2.0 mmol) and ethyl cyanoacetate (2.26 g, 20.0 mmol) was heated in an oil bath at 180 °C for 30 min. The reaction mixture was left to cool, and the solidified mass was triturated with ethyl alcohol, filtered off, dried well, and recrystallized from dimethyl formamide/water (2:1). Yield: 80%; Mp >300 °C. IR (KBr, cm^−1^): 3357 (NH), 2975 (CH), 2265 (CN), 1677 (CO). ^1^H NMR (DMSO-*d_6_*): *δ* 2.34–2.38 (m, 2H, CH_2_), 2.81–2.97 (m, 4H, CH_2_), 4.32 (s, 2H, CH_2_CN), 10.32 (s, 1H, NH). ^13^C NMR (DMSO-*d*_6_): δ 22.51 (CH_2_-Alphatic), 23.14 (cyclopentane C-3), 24.48 (cyclopentane C-4), 29.83 (cyclopentane C-5), 116.87 (CN), 121.50 (pyrimidine C-5), 134.48 (pyrimidine C-6), 136.26 (thiophene C-5), 146.83 (thiophene C-4), 155.07 (triazole C-2), 164.48 (triazole C-5), 171.55 (C=O). Anal. Calcd for C_12_H_9_N_5_OS (271.3): C, 53.13; H, 3.34; N, 25.81; S, 11.82. Found: C, 53.27; H, 3.30; N, 25.58; S, 11.81.

*(E)-2-Amino-3-((4-oxopentan-2-ylidene)amino)-3,5,6,7-tetrahydro-4H-cyclopenta[4,5]thieno[2,3-d]pyrimidin-4-one (* **8** *)*

A mixture of **3** (0.44 g, 2.0 mmol) in acetyl acetone (2.0 g, 20 mmol) was heated under reflux for 45 min and left to cool. The precipitated product was filtered off, washed with diethylether, dried well, and recrystallized from ethanol. Yield: 64%; Mp: >300 °C. IR (KBr, cm^−1^): 3340 (NH_2_), 2995(CH), 1680, 1617 (2C=O), 1601 (C=N).^1^H NMR (DMSO-*d_6_*): *δ* 1.72 (s, 3H, CH_3_), 2.06 (s, 3H, COCH_3_), 2.34–2.38 (m, 2H, CH_2_), 2.56 (s, 2H, CH_2_CO), 2.82–2.84 (t, 2H, CH_2_), 2.91–2.94 (t, 2H, CH_2_), 5.70 (s, 2H, NH_2_). ^13^C NMR (DMSO-*d_6_*): *δ* 14.48 (CH3-Alphatic), 22.26 (cyclopentane C-3), 24.42 (CH_3_CO). 25.50 (cyclopentane C-4), 29.83 (cyclopentane C-5), 48.59 (CH_2-_Aliphatic), 119.83 (pyrimidine C-5), 133.63 (pyrimidine C-6), 135.56 (thiophene C-4), 144.50 (thiophene C-5), 156.92 (triazole C-2), 163.51 (triazole C-5), 170.26,190.05 (C=O). Anal. Calcd for C_14_H_16_N_4_O_2_S (304.37): C, 55.25; H, 5.30; N, 18.41; S, 10.53. Found: C, 54.96; H, 5.22; N, 18.46; S, 10.52.

*2-Thioxo-2,3,7,8-tetrahydro-1H-cyclopenta[4,5]thieno[2,3-d][1,2,4]triazolo[1,5-a]pyrimidin-9(6H)-one (* **9** *)*

Carbon disulphide (1.2 mL, 20 mmol) was added dropwise over a period of 15 min to a mixture of **3** (0.44 g, 2.0 mmol) and sodium ethoxide (4.0 mmol) in absolute ethanol (15 mL). After complete addition, the reaction mixture was stirred at room temperature for 1 h followed by heating under reflux for 24 h. The solvent was evaporated in vacuo. Water was added to the residue and the alkaline solution was filtered. The clear filtrate was acidified with dilute HCl and the separated solid was collected and recrystallized from dimethylformamide. Yield: 63%; Mp: >300 °C. IR (KBr, cm^−1^): 3345 (NH), 2361 (SH), 1679 (C=O), 1611 (C=N). ^1^H NMR (DMSO-*d_6_*): δ 2.36–2.42 (m, 2H, CH_2_), 2.86–2.91 (m, 4H, CH_2_), 10.57, 10.91 (2s, 2H, 2NH). ^13^C NMR (DMSO-*d*_6_): *δ* 24.18 (cyclopentane C-3), 28.56 (cyclopentane C-4), 29.26 (cyclopentane C-5), 122.76 (pyrimidine C-5), 134.63 (thiophene C-4), 142.85 (thiophene C-5), 145.70 (pyrimidine C-6), 163.94 (triazole C-5), 167.59 (C=O), 183.09 (C=S). MS (*m*/*z*, %): 262 (M-2, 35.1), 234 (48.5), 192 (100.0), 165 (29.6), 136 (17.0). Anal. Calcd for C_10_H_8_N_4_OS_2_ (264.33): C, 45.44; H, 3.05; N, 21.20; S, 24.26. Found: C, 45.19; H, 3.25; N, 21.1; S, 24.27.

*2-(4-Aryl)-7,8-dihydro-3H-cyclopenta[4,5]thieno[2,3-d][1,2,4]triazolo[1,5-a]pyrimidin-9(6H)-one (***10a**–**e***)*

*General procedure:* A mixture of **3** (0.44 g, 2.0 mmol) and appropriate aromatic aldehyde (4.0 mmol) was heated in an oil bath at 180 °C for 30 min. The reaction mixture was left to cool, and the solidified mass was triturated with ethyl alcohol, filtered off, dried well and recrystallized from the proper solvent.

*2-(4-Chlorophenyl)-7,8-dihydro-3H-cyclopenta[4,5]thieno[2,3-d][1,2,4]triazolo[1,5-a]pyrimidin-9(6H)-one (* **10a** *)*

Yield: 55%; Mp: 220–222 °C. IR (KBr, cm^−1^): 3342(NH), 2985 (CH), 1680 (C=O), 1612 (C=N). ^1^H NMR (DMSO-*d*_6_): *δ* 2.31–2.34 (m, 2H, CH_2_), 2.80–2.89 (m, 4H, CH_2_), 7.35–7.37 (d, *J = 3.8 Hz*, 2H, Ar-H), 7.64–7.66 (d, *J = 3.8 Hz*, 2H, Ar-H), 10.31 (s, 1H, NH).

MS (*m*/*z*, %): 344 (M^+1^, 10.9), 329 (23.3), 206 (15.0), 192 (100.0), 164 (50.1), 136 (12.8). Anal. Calcd for C_16_H_11_ClN_4_OS (342.8): C, 56.06; H, 3.23; N, 10.34; S, 9.35. Found: C, 56.26; H, 2.11; N, 10.42; S, 9.37.

*2-(4-Bromophenyl)-7,8-dihydro-3H-cyclopenta[4,5]thieno[2,3-d][1,2,4]triazolo[1,5-a]pyrimidin-9(6H)-one (* **10b** *)*

Yield: 63%; Mp: 268–270 °C. IR (KBr, cm^−1^): 3308 (NH), 2982 (CH), 1666 (C=O), 1601 (C=N). ^1^H NMR (DMSO-*d*_6_): *δ* 2.32–2.39 (m, 2H, CH_2_), 2.83–2.91 (m, 4H, CH_2_), 7.11–7.13 (d, *J = 3.8 Hz*, 2H, Ar-H), 7.56–7.58 (d, *J = 3.8 Hz*, 2H, Ar-H), 10.30 (s, 1H, NH). ^13^C NMR (DMSO-*d_6_*): *δ* 23.03 (cyclopentane C-3), 28.03 (cyclopentane C-4), 29.97 (cyclopentane C-5), 121.26 (pyrimidine C-5), 124.42–131.70 (Ar-C), 135.63 (thiophene C-4), 143.50 (thiophene C-5), 144.83 (pyrimidine C-6), 148.43 (Ar-C-1), 162.66 (triazole C-5), 170.97 (C=O). MS (*m*/*z*, %): 390 (M^+2^, 22.6), 221 (12.1), 207 (100.0), 192 (92.3), 165 (69.4), 135 (29.0). Anal. Calcd C_16_H_11_BrN_4_OS (387.25): C, 49.63; H, 2.86; N, 14.47; S, 8.28. Found: C, 49.61; H, 2.66; N, 14.39; S, 8.30.

*2-(4-Nitrophenyl)-3,6,7,8-tetrahydro-9H-cyclopenta[4,5]thieno[2,3-d][1,2,4]triazolo[1,5-a]pyrimidin-9-one (* **10c** *)*

Yield: 70%; Mp: 220 °C. IR (KBr, cm^−1^): 3318 (NH), 2951 (CH), 1658 (C=O), 1613 (C=N). ^1^H NMR (DMSO-*d*_6_): *δ* 2.31–2.38 (m, 2H, CH_2_), 2.80–2.89 (m, 4H, 2CH_2_), 7.22 (d, *J =* 7.2 Hz, 2H, Ar-H), 7.45 (d, *J =* 7.2 Hz, 2H, Ar-H), 10.33 (s, 1H, NH). ^13^C NMR (DMSO-*d_6_*): *δ* 23.61, 27.82, 29.24, 123.05, 125.02, 128.86, 130.91, 134.84, 138.90, 147.59, 149.98, 154.80, 159.95, 169.39. Anal. Calcd for C_16_H_11_N_5_O_3_S (353.36): C, 54.39; H, 3.14; N, 19.82; S, 9.07. Found: C, 54.38; H, 3.12; N, 19.82; S, 9.09.

*2-(4-Methoxyphenyl)-7,8-dihydro-3H-cyclopenta[4,5]thieno[2,3-d][1,2,4]triazolo[1,5-a]pyrimidin-9(6H)-one (* **10d** *)*

Yield: 71%; Mp: 180–182 °C. IR (KBr, cm^−1^): 3396 (NH), 2991 (CH), 1676 (C=O), 1609 (C=N). ^1^H NMR (DMSO-*d*_6_): *δ* 2.30–2.33 (m, 2H, CH_2_), 2.79–2.88 (t, 4H, CH_2_), 3.84 (s, 3H, OCH_3_), 7.19–7.21 (d, *J = 3.8 Hz*, 2H, Ar-H), 7.55–7.57 (d, *J = 3.8Hz*, 2H, Ar-H), 10.34 (s, 1H, NH). ^13^C NMR (DMSO-*d_6_*): *δ* 23.53 (cyclopentane C-3), 28.11 (cyclopentane C-4), 29.97 (cyclopentane C-5), 55.33 (OCH_3_), 114.73–130.11 (Ar-C), 122.67 (pyrimidine C-5), 139.15 (thiophene C-4), 131.60 (thiophene C-5), 139.15 (triazole C-2), 149.53 (triazole C-5), 154.33 (pyrimidine C-6), 163.25 (Ar-C-1), 169.66 (C=O). Anal. Calcd for C_17_H_14_N_4_O_2_S (338.38): C, 60.34; H, 4.17; N, 16.56; S, 9.48. Found: C, 60.51; H, 4.41; N, 16.49; S, 9.47.

*2-(Anthracen-9-yl)-3,6,7,8-tetrahydro-9H-cyclopenta[4,5]thieno[2,3-d][1,2,4]triazolo[1,5-a]pyrimidin-9-one (* **10e** *)*

Yield: 85%; Mp: 101–103 °C. IR (KBr, cm^−1^): 3347 (NH), 3062 (CH-Aromatic), 2921 (CH), 1660 (C=O), 1615 (C=N). ^1^H NMR (DMSO-*d*_6_): *δ* 2.33–2.40 (m, 2H, CH_2_), 2.83–2.98 (m, 4H, CH_2_), 7.25–7.27 (d, *J = 3.8 Hz*, 2H, Ar-H), 7.63–7.64 (d, *J = 3.8 Hz*, 2H, Ar-H), 8.58 (s, 1H, Ar-H), 10.78 (s, 1H, NH). Anal. Calcd for C_24_H_16_N_4_OS (408.48): C, 70.57; H, 3.95; N, 13.72; S, 7.85. Found: C, 70.27; H, 4.16; N, 13.82; S, 7.84

#### 3.2.1. Preparation of Unprotected saccharide-thieno[3,2-d]pyrimidine (**11a**–**d**)

*General procedure:* A mixture of 3 (0.44 g, 2.0 mmol), monosaccharide (2.0 mmol) and iodine (0.005 g, 0.02 mmol) in AcOH (50 mL) was stirred at room temperature in open air. The reaction was completed in 6–12 h as indicated by the TLC analysis. The mixture was triturated with EtOAc to give precipitates which were collected by filtration.

*2-((1S,2R,3R,4R)-1,2,3,4,5-pentahydroxypentyl)-1,6,7,8-tetrahydro-9H-cyclopenta[4,5]thieno[2,3-d][1,2,4]triazolo[1,5-a]pyrimidin-9-one (* **11a** *)*

Recrystallized from ethanol as a Brown solid; Yield 73%; Mp: >300 °C. IR spectrum (KBr, ν, cm^−1^): 3465–3410 (OH), 3430 (NH), 2924 (CH), 1669 (C=O), 1615 (C=N). ^1^H NMR (DMSO-*d*_6_): δ 2.32–2.37 (m, 2H, CH_2_), 2.80–2.89 (m, 4H, 2CH_2_), 3.24 (t, *J =* 9.2 Hz, 1H), 3.35–3.50 (m, 3H), 3.63–3.74 (m, 2H), 4.72 (brs, 1H, OH), 5.08–5.20 (brs, 4H, 4OH), 5.63 (d, *J* = 9.2 Hz, 1H), 10.15 (s, 1H, NH). ^13^C NMR (DMSO-*d_6_*): *δ* 23.61, 27.85, 29.24, 64.14, 70.01, 71.44, 71.65, 72.08, 122.56, 130.10, 139.10, 148.48, 154.63, 159.92, 169.88. Anal. Calcd for C_15_H_18_N_4_O_6_S (382.39): C, 47.12; H, 4.74; N, 14.65; S, 8.38; Found C, 47.14; H, 4.75; N, 14.65; S, 8.37.

*2-((1S,2R,3S,4R)-1,2,3,4,5-pentahydroxypentyl)-1,6,7,8-tetrahydro-9H-cyclopenta[4,5]thieno[2,3-d][1,2,4]triazolo[1,5-a]pyrimidin-9-one (* **11b** *)*

Recrystallized from ethanol as a yellow powder; Yield 75%; Mp: >300 °C. IR spectrum (KBr, ν, cm^−1^): 3436- 3300 (OH), 3317 (NH), 2924 (CH), 1653 (C=O). ^1^H NMR (400 MHz, DMSO-*d_6_*): *δ* 2.31–2.36(m, 2H, CH_2_), 2.80–2.89 (m, 4H, 2-CH_2_), 3.23 (t, *J* = 8.8 Hz, 1H), 3.35–3.46 (m, 3H), 3.63–3.74 (m, 2H), 4.66 (d, *J* = 5.6 Hz, 1H, OH), 4.73 (t, *J* = 5.6 Hz, 1H, OH), 5.04 (d, *J* = 5.6 Hz, 1H, OH), 5.23 (d, *J* = 6 Hz, 1H, OH), 5.64 (d, *J* = 8.8 Hz, 1H), 10.35 (s, 1H, NH). ^13^C NMR (DMSO-*d_6_*): *δ* 23.65 (cyclopentane C-3), 28.03 (cyclopentane C-4), 29.67 (cyclopentane C-5), 63.11 (C-6′), 68.25 (C-4′), 71.26 (C-5′), 72.05 (C-2′), 73.90 (C-3′), 121.33 (pyrimidine C-5), 133.11 (thiophene C-4), 135.15 (pyrimidine C-6), 141.03 (thiophene C-5), 162.68 (triazole C-5), 164.45 (C-1′), 167.66 (C=O). Anal. Calcd for C_15_H_18_N_4_O_6_S (382.39): C, 47.12; H, 4.74; N, 14.65; S, 8.38. Found C, 47.13; H, 4.74; N, 14.65; S, 8.36.

*2-((1R,2R,3R,4R)-1,2,3,4,5-pentahydroxypentyl)-1,6,7,8-tetrahydro-9H-cyclopenta[4,5]thieno[2,3-d][1,2,4]triazolo[1,5-a]pyrimidin-9-one (* **11c** *)*

Recrystallized from ethanol as a yellow powder; Yield 71%; Mp: >300 °C. IR spectrum (KBr, ν, cm^−1^): 3446–3380 (OH), 3416 (NH), 2927 (CH), 1657 (C=O). ^1^H NMR (DMSO-*d_6_*): *δ* 2.32–2.37 (m, 2H, CH_2_), 2.52–2.54 (t, 2H, CH_2_), 2.93–2.95 (t, 2H, CH_2_), 3.36–3.42 (m, 2H, H-6′,6″), 3.52–3.61 (m, 2H, H-4′, H-5′), 3.71–3.78 (dd, 1H, *J* = 2.2 Hz, *J* = 5.8 Hz, H-3′), 3.85–3.93 (dd, 1H, *J* = 2.2 Hz, *J* = 5.6 Hz, H-2′), 4.10–4.17 (m, 2H, 2OH), 4.23–4.31 (d, 1H, *J* = 5.5 Hz, OH), 5.01 (m, 2H, 2OH), 6.13–6.22 (d, *J* = 9.6 Hz, 1H, H-1′), 10.73 (s, 1H, NH). Anal. Calcd for C_15_H_18_N_4_O_6_S (382.39): C, 47.12; H, 4.74; N, 14.65; S, 8.38. Found C, 47.13; H, 4.75; N, 14.65; S, 8.37.

*2-((1R,2S,3R)-1,2,3,4-tetrahydroxybutyl)-1,6,7,8-tetrahydro-9H-cyclopenta[4,5]thieno[2,3-d][1,2,4]triazolo[1,5-a]pyrimidin-9-one (* **11d** *)*

Recrystallized from ethanol as a yellow powder; Yield 69%; Mp: >300 °C. IR spectrum (KBr, ν, cm^−1^): 3410 –3300 (OH), 3355 (NH), 2965 (CH), 1667 (C=O), 1618 (C=N). ^1^H NMR (DMSO-*d_6_*): *δ* 2.31–2.37 (m, 2H, CH_2_), 2.83–2.89 (m, 4H), 3.46–3.51 (m, 1H), 3.55–3.61 (m, 1H), 3.94 (dd, *J* = 8.0 Hz, *J* = 4.0 Hz, 1H), 4.10 (dd, *J* = 9.4 Hz, *J* = 4.8 Hz, 1H), 4.32 (dd, *J* = 10.6 Hz, *J* = 4.8 Hz, 1H), 4.98 (t, *J* = 5.2 Hz, 1H, OH), 5.21 (d, *J* = 5.2 Hz, 1H, OH), 5.51 (d, *J* = 6.4 Hz, 1H, OH), 5.80 (d, *J* = 4.8 Hz, 1H), 11.11 (s, 1H, NH). ^13^C NMR (DMSO-*d*_6_): *δ* 23.60, 27.82, 29.26, 62.91, 70.37, 72.52, 74.37, 122.54, 130.11, 139.12, 148.45, 154.60, 159.93, 169.86. Anal. Calcd for C_14_H_16_N_4_O_5_S (352.37): C, 47.72; H, 4.58; N, 15.90; S, 9.10. Found C, 47.73; H, 4.61; N, 15.90; S, 9.12

#### 3.2.2. Acetylation of saccharide-thieno[3,2-d]pyrimidine (**11a**–**d**)

*General procedure:* To a solution of glycosides (**11a–d**) (1 mmol) in pyridine (15 mL) acetic anhydride (5 mmol) was added and obtained clear solution was stirred at room temperature for 10 h. The reaction mixture was poured onto crushed ice, and the product that separated out was filtered off, washed with sodium hydrogen carbonate and water then dried well and recrystallized from ethyl acetate to give the acetylated products (**12a–d**).

*(1S,2R,3R,4R)-1-(9-oxo-1,7,8,9-tetrahydro-6H-cyclopenta[4,5]thieno[2,3-d][1,2,4]triazolo[1,5-a]pyrimidin-2-yl)pentane-1,2,3,4,5-pentayl pentaacetate (* **12a** *)*

Recrystallized from ethanol as a yellow powder; Yield 70%; Mp: >300 °C. IR spectrum (KBr, ν, cm^−1^): 3335 (NH), 2957 (CH) 1745, 1632 (2C=O). ^1^H NMR (DMSO-*d6*): δ 1.85, 1.95, 2.05, 2.14, 2.18 (5s, 15H, 5CH3), 2.31–2.34 (m, 2H, CH_2_), 2.81–2.86 (t, 2H, CH2), 2.93–2.97 (t, 2H, CH_2_), 3.90–4.05 (m, 2H, H-6′,6″), 4.15–4.20 (m, 1H, H-5′), 5.05–5.10 (m, 2H, H-4′, H-3′), 5.38–5.42 (dd, 1H, *J* = 2.2 Hz, *J* = 5.6 Hz, H-2′), 7.15- 7.17 (d, *J* = 9.6 Hz, 1H, H-1′), 12.03 (s,1H, NH). Anal. Calcd for C_25_H_28_N_4_O_11_S (592.58): C, 50.67; H, 4.76; N, 9.45; S, 5.41. Found C, 50.68; H, 4.76; N, 9.45; S, 5.40.

*(1S,2R,3S,4R)-1-(9-oxo-1,7,8,9-tetrahydro-6H-cyclopenta[4,5]thieno[2,3-d][1,2,4]triazolo[1,5-a]pyrimidin-2-yl)pentane-1,2,3,4,5-pentayl pentaacetate (* **12b** *)*

Recrystallized from ethanol as a yellow powder; Yield 68%; Mp: >300 °C. IR spectrum (KBr, ν, cm^−1^): 3429 (NH), 2923 (CH), 1750, 1633 (2C=O), 1613 (C=N); ^1^H NMR (CDCl_3_): δ 1.95, 2.00, 2.05, 2.12, 2.19 (5s, 15H, 5CH_3_), 2.31–2.38 (m, 2H, CH2), 2.80–2.88 (m, 4H, CH_2_), 4.16 (dd, *J* = 12.6, *J* = 1.8 Hz, 1H), 4.33 (dd, *J* = 12.6, *J* = 5.0 Hz, 1H), 4.47–4.50 (m, 1H), 5.54 (dd, 1H, *J* = 12.2 Hz, *J* = 4.2 Hz, 1H), 5.95 (t, *J* = 9.02 Hz), 6.34(d, *J* = 9.2 Hz, 1H), 11.91(s, 1H, NH). ^13^C NMR (DMSO-*d_6_*): δ 20.04, 20.94, 21.12, 21.29, 21.52 (5CH_3_), 25.02(cyclopentane C-3), 26.21 (cyclopentane C-4), 32.32 (cyclopentane C-5), 60.92(C-6′), 63.02 (C-5′), 69.51 (C-4′), 74.94 (C-3′), 75.42 (C-2′), 119.54 (pyrimidine C-4), 128.12 (thiophene C-4), 134.08 (thiophene C-5), 145.90 (C-1′), 157.42 (pyrimidine C-5), 164.74 (triazole C-4), 167.91 (triazole C-1), 168.51, 170.10, 170.50, 171.17, 171.66, 172.49, (6 C=O). Anal. Calcd for C_25_H_28_N_4_O_11_S (592.58): C, 50.67; H, 4.76; N, 9.45; S, 5.41. Found C, 50.66; H, 4.75; N, 9.45; S, 5.41.

*1R,2R,3R,4R)-1-(9-oxo-1,7,8,9-tetrahydro-6H-cyclopenta[4,5]thieno[2,3-d][1,2,4]triazolo[1,5-a]pyrimidin-2-yl)pentane-1,2,3,4,5-pentayl pentaacetate (* **12c** *)*

Recrystallized from ethanol as a yellow powder; Yield 71%; Mp: >300 °C. IR spectrum (KBr, ν, cm^−1^): 3432 (NH), 2933 (CH) 1742, 1642 (2C=O); ^1^H NMR (DMSO-*d*_6_): δ 1.95, 2.05, 2.10, 2.14, 2.17 (5s, 15H, 5CH_3_), 2.30–2.35 (m, 2H, CH_2_), 2.82–2.87 (t, 2H, CH_2_), 2.93–2.97 (t, 2H, CH_2_), 4.04–4.11 (m, 2H, H-6′,6″), 4.50–4.58 (m, 1H, H-5′), 5.15–5.23 (m, 2H, H-4′, H-3′), 5.50–5.56 (dd, 1H, *J* = 2.1 Hz, *J* = 5.9 Hz, H-2′), 7.05–7.12 (d, *J* = 9.2 Hz, 1H, H-1′), 11.53 (s,1H, NH). Anal. Calcd for C_25_H_28_N_4_O_11_S (592.58): C, 50.67; H, 4.76; N, 9.45; S, 5.41. Found C, 50.68; H, 4.76; N, 9.45; S, 5.43.

*(1R,2S,3R)-1-(9-oxo-1,7,8,9-tetrahydro-6H-cyclopenta[4,5]thieno[2,3-d][1,2,4]triazolo[1,5-a]pyrimidin-2-yl)butane-1,2,3,4-tetrayl tetraacetate (* **12d** *)*

Recrystallized from ethanol as a brown powder; Yield 65%; Mp: > 300 °C. IR spectrum (KBr, ν, cm^−1^): 3438 (NH), 2963 (CH), 1751, 1634 (2C=O); ^1^H NMR (CDCl_3_): *δ* 1.88, 2.04, 2.07, 2.08 (4s, 12H, 4CH_3_), 2.33–2.36 (m, 2H, CH_2_), 2.90–2.98 (m, 4H, 2CH_2_), 4.23 (dd, *J* = 12.4 Hz, *J* = 4.0 Hz, 1H), 4.48 (dd, *J* = 8.2 Hz, *J* = 4.2 Hz, 1H), 5.63 (t, *J* = 5.4 Hz, 1H), 5.09 (dd, *J* = 4.8 Hz, *J* = 4.0 Hz, 1H), 6.32 (d, *J* = 3.6 Hz, 1H), 11.78 (s,1H, NH). ^13^C NMR (CDCl_3_): *δ* 20.10, 20.43, 20.47, 20.59 (4 CH_3_), 23.01 (cyclopentane C-3), 27.81 (cyclopentane C-4), 29.98 (cyclopentane C-5), 64.28, 68.43, 70.43, 71.54, 122.62, 130.09, 139.07, 148.48, 155.04, 160.02, 168.71, 169.05, 169.36, 169.89, 170.50 (5 C=O). Anal. Calcd for C_22_H_24_N_4_O_9_S (520.51): C, 50.77; H, 4.65; N, 10.76; S, 6.16; Found C, 50.79; H, 4.66; N, 10.76; S, 6.18.

#### 3.2.3. Glycosylation Procedures of Compound (**14–16**)

*General procedure:* Potassium hydroxide (12 mmol) suspended in water (1 mL) was added to a well-stirred solution of the thienopyrimidine derivatives **4** or **8** or **10d** (10 mmol) in acetone (15 mL). α-bromocetoglucose (12 mmol) dissolved in acetone (10 mL) was added and the reaction mixture was stirred at r.t for 18 h (TLC: Pet. ether/ethyl acetate; 4:1). The solvent was evaporated, and the residue was treated with pet. ether (40–60, 2 × 15 mL) to form a solid which was filtered and dried to give compounds **14**–**16**.

*3-(2,3,4,6-Tetra-O-acetyl-β-d-glocopyranosyl)-7,8-dihydro-3H-cyclopenta-[4,5]thieno-[2,3-d][1,2,4]triazolo[1,5-a]pyrimidin-9(6H)-one (* **14** *)*

Yield: 73%; Mp: >300 °C. IR spectrum (KBr, ν, cm^−1^): 2930 (CH), 1741, 1655 (2C=O);

^1^H NMR (CDCl_3_): δ 1.98, 2.00, 2.05, 2.07 (4s, 12H, CH_3_CO), 2.31–2.38 (m, 2H, CH_2_), 2.80–2.88 (m, 4H, 2CH_2_), 3.76–3.80 (m, 1H), 4.14 (dd, J = 12.4, J = 2.5 Hz, 1H), 4.25 (dd, J = 12.4, J = 4.8 Hz, 1H), 4.63 (d, 1H, *J* = 8.8 Hz,), 4.92 (t, J = 9.2 Hz, 1H), 5.07 (t, J = 10 Hz, 1H), 5.19 (t, J = 9.2 Hz, 1H), 7.55 (s, 1H, triazole-CH). ^13^C NMR (DMSO-*d_6_*): *δ* 20.03, 20.08, 20.11, 20.55 (4CH_3_), 24.59(cyclopentane C-3), 28.03 (cyclopentane C-4), 29.11 (cyclopentane C-5), 69.66(C-6′), 69.94 (C-5′), 71.05 (C-4′), 72.80 (C-3′), 73.09 (C-2′), 85.83 (C-1′), 128.56 (thiophene C-4), 133.86 (pyrimidine C-6), 141.59 (thiophene C-5), 147.53 (pyrimidine C-5), 156.83 (triazole C-5), 161.90 (triazole C-2), 170.01, 170.86, 171.11, 172.51, 173.91, (5C=O). Anal. Calcd for C_24_H_26_N_4_O_10_S (562.55): C, 51.24; H, 4.66; N, 9.96; S, 5.70. Found: C, 51.05; H, 4.60; N, 9.88; S, 5.71.

*(E)-3-((4-Oxopentan-2-ylidene)amino)-2-((2,3,4,6-tetra-O-acetyl-β-D-glocopyranosyl)amino)-6,7-dihydro-3H-cyclopenta[4,5]thieno[2,3-d]pyrimidin-4(5H)-one (* **15** *)*

Yield: 62%; Mp: >300 °C. IR spectrum (KBr, ν, cm^−1^): 3334 (NH), 3072 (C-H), 1752, 1662, 1640 (2C=O), 1610 (C=N); ^1^H NMR (DMSO-*d_6_*): *δ* 1.70 (s, 3H, CH_3_), 1.82, 1.99, 2.08, 2.12, 2.21 (5s, 15H, 5CH_3_CO), 2.33–2.38 (m, 2H, CH_2_), 2.81–2.88 (t, 2H, CH_2_), 2.91–2.98 (t, 2H, CH_2_), 3.18 (s, 2H, CH_2_), 3.99–4.05 (dd, 1H, *J* = 3.6, *J* = 10.6 Hz, H-6′), 4.14–4.21 (dd, 1H, *J* = 3.6, *J* = 10.6 Hz, H-6″), 4.41–4.46 (m, 1H, H-5′), 4.66–4.71 (m, 1H, H-4′), 5.23–5.25 (d, 1H, *J* = 10.3 Hz, H-1′), 5.31–5.36 (dd, 1H, *J* = 8.8, *J* = 9.7 Hz, H-2′), 6.07–6.11 (t, 1H, *J* = 8.2Hz, H-3′). Anal. Calcd for C_28_H_34_N_4_O_11_S (634.65): C, 52.99; H, 5.40; N, 8.83; S, 5.05. Found: C, 52.86; H, 5.39; N, 8,65; S, 5.20.

*2-(4-Methoxyphenyl)-3-(2,3,4,6-tetra-O-acetyl-β-D-glocopyranosyl)-7,8-dihydro-3H-cyclopenta[4,5]thieno[2,3-d][1,2,4]triazolo[1,5-a]pyrimidin-9(6H)-one (* **16** *)*

Yield: 65%; Mp: >300 °C. IR spectrum (KBr, ν, cm^−1^): 2932 (C-H), 1719, 1632 (2C=O); ^1^H NMR (CDCl_3_): δ 1.98, 2.00, 2.05, 2.07 (4s, 12H, CH_3_CO), 2.32–2.39 (m, 2H, CH_2_), 2.80–2.89 (m, 4H, 2CH_2_), 3.76–3.80 (m, 1H), 3.88 (s, 3H, OCH_3_), 4.14 (dd, 1H, *J* = 12.4, J = 2.0 Hz, 1H), 4.25 (dd, 1H, *J* = 12.4, *J* = 4.8 Hz, 1H), 4.63 (d, *J* = 8.8 Hz, 1H), 4.93 (t, *J* = 9.2 Hz, 1H), 5.08 (t, *J* = 9.6 Hz, 1H), 5.19 (t, *J* = 9.2, 1H), 7.35 (d, *J* = 7.6 Hz, 2H, Ar-H), 7.53 (d, *J* = 7.6 Hz, 2H, Ar-H). ^13^C NMR (CDCl_3_): *δ* 20.14, 20.51, 20.65, 23.12, 27.83, 29.94 (cyclopentane C-5), 55.30 (OCH_3_), 61.56, 67.74, 70.19, 72.72, 75.16, 85.79, 117.69, 125.89, 128.55, 128.85, 129.87, 139.15, 148.48, 154.30, 160.02, 163.22, 168.98, 169.35, 169.62, 169.88, 170.46 (5 C=O). Anal. Calcd for C_31_H_32_N_4_O_11_S (668.67): C, 55.68; H, 4.82; N, 8.38; S, 4.79; Found: C, 55.61; H, 4.63; N, 8.58; S, 4.80.

### 3.3. In Vitro Cytotoxic Screening

The cell lines were obtained from Karolinska Center, Department of Oncology and Pathology, Karolinska Institute and Hospital, Stockholm, Sweden. as follows: human breast MCF-7, colorectal HCT-116 and prostate PC-3 cancer cell lines and human skin normal BJ-1 cell line. Exponentially, cells were cultured at a concentration of 10^4^ cells/well for 24 h, afterwards fresh medium containing different concentrations of the tested samples was added. Serial two-fold dilution of the tested samples were added using a multichannel pipette. Moreover, all cells were cultivated at 37 °C, 5% CO_2_ and 95% humidity. Also, incubation of control cells occurred at 37 °C. However, after incubation for 24 h different concentrations of samples (100, 50, 25 and 12.5 µM) were added and the incubation was continued for 48 h, then crystal violet solution 1% was added to each well for 0.5 h to examine the presence of viable cells. After rinsing the wells using water until stain free, 30% glacial acetic acid was added to all wells with shaking the plates on a Microplate reader (TECAN, Inc.) to measure the absorbance at a wavelength of 490 nm. The cytotoxicity was estimated by IC_50_ in μM, which is the concentration that inhibits 50% of growth of cancer cells.

### 3.4. Molecular Docking Study

Molecular docking simulation of the promising in vitro screened cyclopenta[4,5]thieno[2,3-*d*][1,2,4]triazolo[1,5-*a*]pyrimidin-9(6*H*)-ones **10b** and **10e** against EGFR and PI3K was done using the Molecular Operating Environment software (MOE-Dock) version 2014.01. The co-crystallized structures of EGFR and PI3K kinases complexed with their native ligands, erlotinib and quinolone LXX, were downloaded from the protein data bank (PDB codes: 1M17 and 3L54, respectively). All minimizations were performed using MOE until an RMSD gradient of 0.05 kcal∙mol^−1^Å^−1^ with MMFF94x force field and the partial charges were automatically calculated. Preparation of the enzyme structures was done for molecular docking using Protonate 3D protocol with the default options in MOE. London dG scoring function and Triangle Matcher placement method were used in the docking protocol. Initially, the original ligands were re-docked into the active binding site of EGFR and PI3K kinases to assess the root-mean-square deviation values. Thereafter, docking of the newly targeted compounds was performed within the ATP-binding sites of both target kinases after elimination of the co-crystallized ligands.

### 3.5. ADME Prediction

Investigating the absorption, distribution, metabolism, and excretion (ADME) is a critical first step in selecting the best candidates. SwissADME, a free online tool, was used for prediction of the best two compounds’ ADME characteristics [48,49,50]. Veber’s rule (molecule with number of rotatable bonds ≤ 10, TPSA ≤ 140 Å^2^) and Lipinski’s rule (MW ≤ 500, MLogP ≤ 4.15, number of hydrogen bond acceptors ≤ 10 and number of hydrogen bond donors ≤ 5) should be taken into consideration while selecting an oral drug. Anthracenyl derivative **10e** had one violation with MLogP > 4.15, whereas 4-bromophenyl **10b** seemed to be in accordance with the prior rules with no violations (Table 2).

## 4. Conclusions

In this study a series of new thieno[3,2-*d*]pyrimidin-4(3*H*)-one derivatives as anti-cancer agents was designed and synthesized. The compounds were tested against three cancer cell lines, namely, MCF-7, HCT-116, and PC-3. Compounds **10b** and **10e** displayed superior and excellent cytotoxicity against MCF-7 cell lines (IC_50_ = 19.4 ± 0.22 and 14.5 ± 0.30 μM, respectively) in comparison to doxorubicin. Both derivatives showed promising drug-like characteristics and docking simulation results against EGFR and PI3K. Thus, **10b** and **10e** can be considered as lead compounds for this series that merit further development efforts to obtain potent anticancer candidates.

## Figures and Tables

**Table 1:** The antitumor activities of the target compounds against MCF-7, HCT-116, PC-3, and BJ-1 cancer cell lines expressed as IC_50_ values.

Compound No.	IC_50_ (Mean ± SD) (µM)			
	MCF-7	HCT-116	PC-3	BJ-1
**9**	71.8 ± 1.05	–	–	–
**10a**	60.6 ± 0.45	–	–	–
**10b**	19.4 ± 0.22	–	–	221.7 ± 30
**10e**	14.5 ± 0.30	57.01 ± 0.61	25.23 ± 0.40	34.81 ± 4.5
**Doxorubicin**	40.0 ± 3.9	20.5 ± 2.1	6.8 ± 1.2	49.25 ± 1.08

IC_50_: Compound concentration required to inhibit growth by 50%, SD: standard deviation; each value is the mean of three values, (–) not detected.

**Table 2:** Predicted physicochemical properties of cyclopenta[4,5]thieno[2,3-*d*][1,2,4]triazolo[1,5-*a*]pyrimidinones **10b** and **10e**.

Compd. No.	MW ^a^	nHBD ^b^	nHBA ^c^	nRB ^d^	MLogP ^e^	TPSA (Å^2^) ^f^	Violations ^g^
**10b**	387.25	1	3	1	3.73	91.29	0 (Lipinski & Veber)
**10e**	408.48	1	3	1	4.96	91.29	1 (Lipinski), 0 (Veber)

^a^ Molecular weight; ^b^ number of hydrogen bond donor; ^c^ number of hydrogen bond acceptor; ^d^ number of rotatable bond; ^e^ calculated lipophilicity (MLog Po/w); ^f^ topological polar surface area; ^g^ violations from Lipinski and Veber rules.

**Table 3:** Predicted pharmacokinetic properties of cyclopenta[4,5]thieno[2,3-*d*][1,2,4]triazolo[1,5-*a*]pyrimidin-9(6*H*)-ones **10b** and **10e**.

Pharmacokinetic Properties	Compd. No.	
	10b	10e
GIT absorption	High	High
BBB permeability	NO	NO
P-gp substrate	NO	YES
Bioavailability score	0.55	0.55
PAINS alert	0	0

## Data Availability

The data presented in this study are available in the article and Supplementary Materials.

## Supplementary Materials

The following supporting information can be downloaded at: https://www.mdpi.com/article/10.3390/molecules29051067/s1. Charts S1–S29: 1H-NMR and 13C-NMR spectra of sample compounds.
