# Supplementary material for: Designing Potent Anti-Cancer Agents: Synthesis and Molecular Docking Studies of Thieno[2,3-d][1,2,4]triazolo[1,5-a]pyrimidine Derivatives
Source: Molecules. 2024 Feb 29;29(5):1067. doi: 10.3390/molecules29051067 (PMC10934261; doi:10.3390/molecules29051067)
Supplement: Supplementary file 1 [file molecules-29-01067-s001.zip › molecules-2823647-supplementary.pdf]

Article

Designing Potent Anti-Cancer Agents: Synthesis and Molecular Docking Studies of Thieno[2,3-d][1,2,4]triazolo[1,5-a]pyrimidine Derivatives

Eman S. M. Elsenbawy [1], Zafer S. Alshehri [2], Nouf A. Babteen [3], Adel A.-H. Abdel-Rahman [1], Mai A. El-Manawaty [4], Eman S. Nossier [5] Reem K. Arafa [6] and Nasser A. Hassan [7,*]

1 Department of Chemistry, Faculty of Science, Menofia University, Shbien El-Kom 32511, Egypt; eman_alsenbawy@yahoo.com (E.S.M.E.); adelnassar63@yahoo.com (A.A.-H.A.-R.)

2 Department of Medical Laboratories, College of Applied Medical Sciences, Shaqra University, Dawadmi 19257, Saudi Arabia; zaf@su.edu.sa

3 Department of Biochemistry, College of Sciences, Jeddah University, Jeddah 21577, Saudi Arabia; nababteen@uj.edu.sa

4 Department of Pharmacognosy, Pharmaceutical Science Division, National Research Centre, Cairo 12622, Egypt; mayalyem@gmail.com

5 Department of Pharmaceutical Medicinal Chemistry and Drug Design, Faculty of Pharmacy (Girls), Al-Azhar University, Cairo 11754, Egypt; dr.emannossier@gmail.com

6 Drug Design and Discovery Laboratory, Biomedical Sciences Program, University of Science and Technology, Zewail City of Science and Technology, Ahmed Zewail Road, October Gardens, Cairo 12578, Egypt; rkhidr@zewailcity.edu.eg

7 Synthetic Unit, Department of Photochemistry, Chemical Industries Research Institute, National Research Centre, Cairo 12622, Egypt

* Correspondence: nasserabdelhamid@hotmail.com

Supplementary material

1. Experimental

1.1. *In vitro* cytotoxic screening

The cell lines were obtained from Karolinska Center, Department of Oncology and Pathology, Karolinska Institute and Hospital, Stockholm, Sweden. as follows: human breast MCF-7, colorectal HCT-116 and prostate PC-3 cancer cell lines and human skin normal BJ-1 cell line. Exponentially, cells were placed in 10^4 cells/ well for 24 h, and then add fresh medium which containing different concentration of the tested sample. Serial two-fold dilution of the tested sample were added using a multichannel pipette. Moreover, all cells were cultivated at 37 °C, 5% CO_2 and 95% humidity. Also, incubation of control cells occurred at 37 °C. However, after incubation for 24 h different concentrations of sample (100, 50, 25 and 12.5 µM) were added and continued the incubation for 48 h, then, add the crystal violet solution 1% to each well for 0.5 h to examine viable cells. Rinse the wells using water until no stain. After that, add 30% glacial acetic acid to all wells with shaking plates on Microplate reader (TECAN, Inc.) to measure the absorbance, using a test wavelength of 490 nm. Besides, compare the treated

samples with the control cell. The cytotoxicity was estimated by IC_{50} in (μM), the concentration that inhibits 50% of growth of cancer cell.

1.2. In silico studies

1.2.1. Molecular docking study

The molecular docking simulation of the promising *in vitro* screened cyclopenta[4,5]thieno[2,3-d][1,2,4]triazolo[1,5-*a*]pyrimidin-9(6*H*)-ones **10b** and **10e** against EGFR and PI3K was done using the Molecular Operating Environment software (MOE-Dock) version 2014.0901. The co-crystallized structures of EGFR and PI3K kinases complexed with their native ligands, erlotinib and quinolone **LXX**, were downloaded from the protein data bank (PDB codes: 1M17 and 3L54, respectively). All minimizations were performed using MOE until an RMSD gradient of 0.05 $kcal{\cdot}mol^{-1}Å^{-1}$ with MMFF94x force field and the partial charges were automatically calculated. Preparation of the enzyme structures was done for molecular docking using Protonate 3D protocol with the default options in MOE. London dG scoring function and Triangle Matcher placement method were used in the docking protocol. Initially, the original ligands were re-docked into the active binding site of EGFR and PI3K kinases to assess the root-mean-square deviation values. Then, the docking studies of the newly targeted compounds were estimated within the ATP-binding sites after elimination of the co-crystallized ligands.

Percentage cytotoxicity of 100 μM of the compounds on human tumor cell lines*

	HCT-116	PC-3	MCF-7
**4**	9.1±2.0	11.3±3.7	34.8±4.1
**5**	0.0±0.005	0.0±0.005	18.9±10
**6**	0.0±0.005	15.1±4.1	24±2.5
**8**	0.0±0.005	0.0±0.005	45.7±5.9
**9**	0.0±0.005	19.3±3.5	62.5±7.6
**10a**	20.3±2.1	40.9±4.1	61.4±4.7
**10b**	24.4±6.6	41.4±0.7	86.4±10
**10c**	40.4±2.4	54.7±2.8	33.62±2.13
**10d**	14.3±4.3	36.4±3.1	53.1±6.1
**10e**	86.7±4.4	75.9±1.3	98.3±0.5
**11a**	32.4±3.9	33.9±3.5	36.6±6.3
**11b**	18.7±7.6	12.7±3.8	39.4±8.3
**11c**	20.6±5.4	8.5±4.5	38.7±1.5
**11d**	10.0±3.4	25.3±3.8	50.5±0.2
**12b**	13.3±3.1	20.6±3.3	30.1±7.5

**12d**	9.5±1.2	14.1±6.2	26.9±4.8
**14**	15.7±2.9	9.5±1.9	29.2±0.8
**15**	3.0±1.2	5.6±1.2	19.7±3.7
**16**	1.0±0.5	14.5±1.6	29.5±1.6

*The results are shown as average ± standard deviation

Percentage cytotoxicity of 50 μM of the compounds on human tumor cell lines*

	**HCT-116**	**PC-3**	**MCF-7**
4	0.0±0.005	0.0±0.005	35.5±4.4
5	0.0±0.005	0.0±0.005	0.0±0.005
6	0.0±0.005	0.0±0.005	0.0±0.005
8	0.0±0.005	0.0±0.005	40.9±7.8
9	0.0±0.005	7.8±1.3	32.7±2.4
10a	17.9±1.7	35.9±0.8	44.4±11.2
10b	15.0±2.5	22.1±0.6	80.8±2.8
10c	39.4±0.9	54.8±1.6	25.97±1.28
10d	0.0±0.005	26.9±1.2	47.3±8.5
10e	34.9±2.8	64.2±1.1	97.9±1.2
11a	25.8±1.6	7.3±0.3	41.3±4.3
11b	21.8±1.9	0.0±0.005	22.1±6.9
11c	22.3±0.9	0.0±0.005	16.5±2.1
11d	0.0±0.005	9.7±0.5	47.9±2.2
12b	0.0±0.005	7.3±0.9	20.8±2.1
12d	0.0±0.005	0.0±0.005	0.0±0.005
14	0.0±0.005	0.0±0.005	0.0±0.005
15			
16	0.0±0.005	0.0±0.005	0.0±0.005

Percentage cytotoxicity of different concentrations of the compounds on HCT-116 human tumor cell line*

	**100 μM**	**50 μM**	**25 μM**	**12.5 μM**
4	9.1±2.0	0.0±0.005	0.0±0.005	0.0±0.005
5	0.0±0.005	0.0±0.005	0.0±0.005	0.0±0.005
6	0.0±0.005	0.0±0.005	0.0±0.005	0.0±0.005
8	0.0±0.005	0.0±0.005	0.0±0.005	0.0±0.005
9	0.0±0.005	0.0±0.005	0.0±0.005	0.0±0.005
10a	20.3±2.1	17.9±1.7	17.8±1.7	14.4±2.3
10b	24.4±6.6	15.0±2.5	14.3±1.7	6.2±0.9
10c	40.4±2.4	39.4±0.9	38.2±0.9	20.2±2.9
10d	14.3±4.3	0.0±0.005	0.0±0.005	0.0±0.005
10e	86.7±4.4	34.9±2.8	19.0±4.9	0.0±0.005
11a	32.4±3.9	25.8±1.6	12.94±4.6	12.94±4.6
11b	18.7±7.6	21.8±1.9	16.1±1.6	18.2±2.3
11c	20.6±5.4	22.3±0.9	19.5±3.7	12.7±1.7
11d	10.0±3.4	0.0±0.005	0.0±0.005	0.0±0.005

12b	13.3±3.1	0.0±0.005	0.0±0.005	0.0±0.005
12d	9.5±1.2	0.0±0.005	0.0±0.005	0.0±0.005
14	15.7±2.9	0.0±0.005	0.0±0.005	0.0±0.005
15	3.0±1.2	0.0±0.005	0.0±0.005	0.0±0.005
16	1.0±0.5	0.0±0.005	0.0±0.005	0.0±0.005

*The results are shown as average ± standard deviation

Percentage cytotoxicity of different concentrations of the compounds on PC-3 human tumor cell line*

	**100 µM**	**50 µM**	**25 µM**	**12.5 µM**
4	11.3±3.7	0.0±0.005	0.0±0.005	0.0±0.005
5	0.0±0.005	0.0±0.005	0.0±0.005	0.0±0.005
6	15.1±4.1	0.0±0.005	0.0±0.005	0.0±0.005
8	0.0±0.005	0.0±0.005	0.0±0.005	0.0±0.005
9	19.3±3.5	7.8±1.3	6.3±0.9	3.7±1.3
10a	40.9±4.1	35.9±0.8	30.7±0.5	20.7±3.9
10b	41.4±0.7	22.1±0.6	18.4±1.7	10.9±5.2
10c	54.7±2.8	54.8±1.6	53.4±1.2	48.3±2.0
10d	36.4±3.1	26.9±1.2	21.6±0.06	9.1±6.3
10e	75.9±1.3	64.2±1.1	56.6±1.3	29.6±3.8
11a	33.9±3.5	7.3±0.3	21.9±1.0	9.9±2.5
11b	12.7±3.8	0.0±0.005	0.0±0.005	0.0±0.005
11c	8.5±4.5	0.0±0.005	0.0±0.005	0.0±0.005
11d	25.3±3.8	9.7±0.5	6.8±0.3	5.4±6.8
12b	20.6±3.3	7.3±0.9	4.8±1.1	1.5±2.3
12d	14.1±6.2	0.0±0.005	0.0±0.005	0.0±0.005
14	9.5±1.9	0.0±0.005	0.0±0.005	0.0±0.005
15	5.6±1.2	0.0±0.005	0.0±0.005	0.0±0.005
16	14.5±1.6	0.0±0.005	0.0±0.005	0.0±0.005

*The results are shown as average ± standard deviation

Percentage cytotoxicity of different concentrations of the compounds on MCF-7 human tumor cell line*

	100 µM	50 µM	25 µM	12.5 µM
4	34.8±4.1	35.5±4.4	23.6±3.2	31.5±5.7
5	18.9±10	0.0±0.005	0.0±0.005	0.0±0.005
6	24±2.5	0.0±0.005	0.0±0.005	0.0±0.005
8	45.7±5.9	40.9±7.8	31.9±3.1	30.1±4.1
9	62.5±7.6	32.7±2.4	28.4±2.6	0.0±0.005
10a	61.4±4.7	44.4±11.2	33.3±0.8	16.5±9.5
10b	86.4±10	80.8±2.8	64.4±2.2	30.3±4.3
10c	33.62±2.13	25.97±1.28	24.04±2.59	8.18±1.01
10d	53.1±6.1	47.3±8.5	35.1±5.9	12.7±2.1
10e	98.3±0.5	97.9±1.2	85.6±3.6	37.9±6.7
11a	41.3±4.3	36.6±6.3	16.2±1.6	0.0±0.005
11b	39.4±8.3	22.1±6.9	0.0±0.005	0.0±0.005
11c	38.7±1.5	16.5±2.1	0.0±0.005	0.0±0.005
11d	50.5±0.2	47.9±2.2	29.2±2.9	0.0±0.005
12b	30.1±7.5	20.8±2.1	5.5±0.6	0.0±0.005
12d	26.9±4.8	0.0±0.005	0.0±0.005	0.0±0.005
14	29.2±0.8	0.0±0.005	0.0±0.005	0.0±0.005
15	19.7±3.7	0.0±0.005	0.0±0.005	0.0±0.005
16	29.5±1.6	0.0±0.005	0.0±0.005	0.0±0.005

*The results are shown as average ± standard deviation

Percentage cytotoxicity of different concentrations of the active compounds on normal BJ1 human skin fibroblast cell line*

	100 µM	50 µM	25 µM	12.5 µM
**10b**	**39.3±7.7**	**34.6±3.8**	**34.2±7.4**	**16.8±8.3**
**10e**	**83.9±1.2**	**58.1±3.2**	**44.8±1.9**	**13.8±2.1**

*The results are shown as average ± standard deviation

The IC_{50} values (in µM) of the compounds which gave more than 60% at 100µM

Compounds	HCT-116	PC-3	MCF-7	BJ-11
**9**	-	-	**71.8±10, r2=0.91**	-
**10a**	-	-	**60.6±4.5, r2=0.98**	-
**10b**	-	-	**19.4±2, r2=0.96**	**221.7±30, r2=0.97**
**10e**	**57.01±6, r^2=0.96**	**25.23±4, r^2=0.93**	**14.5±0.3, r2=0.99**	**34.81±4.5, r2=0.94**

The results are represented as average IC_{50} ± SD, coefficient of determination

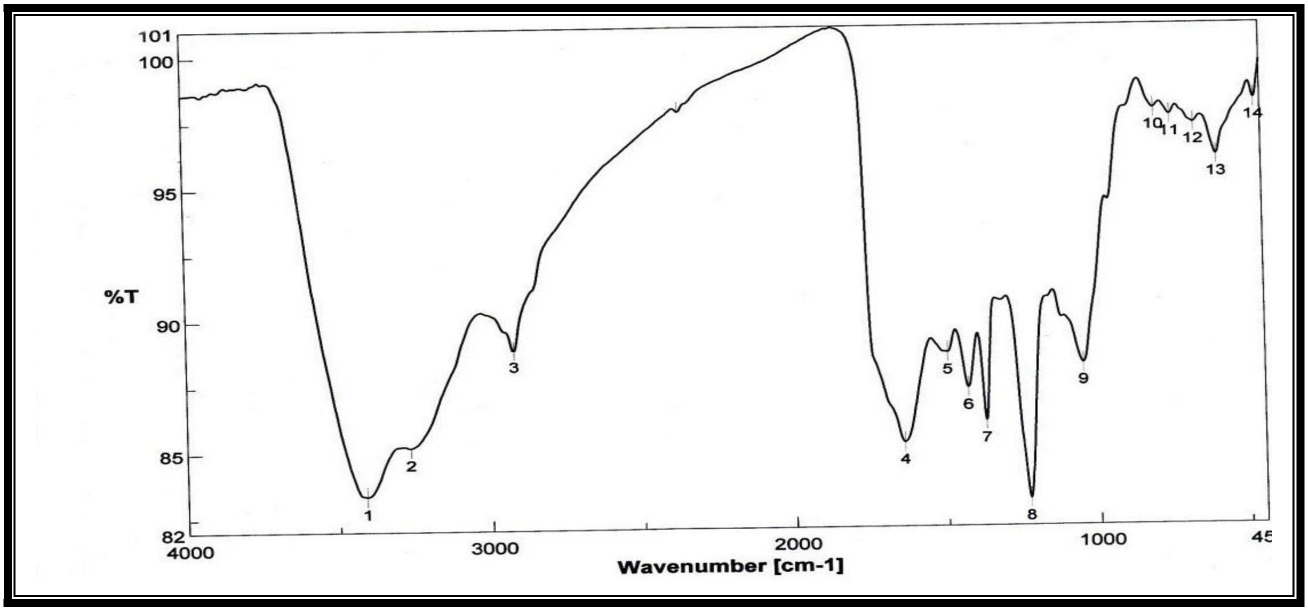

Chart S1: IR of Compound 3

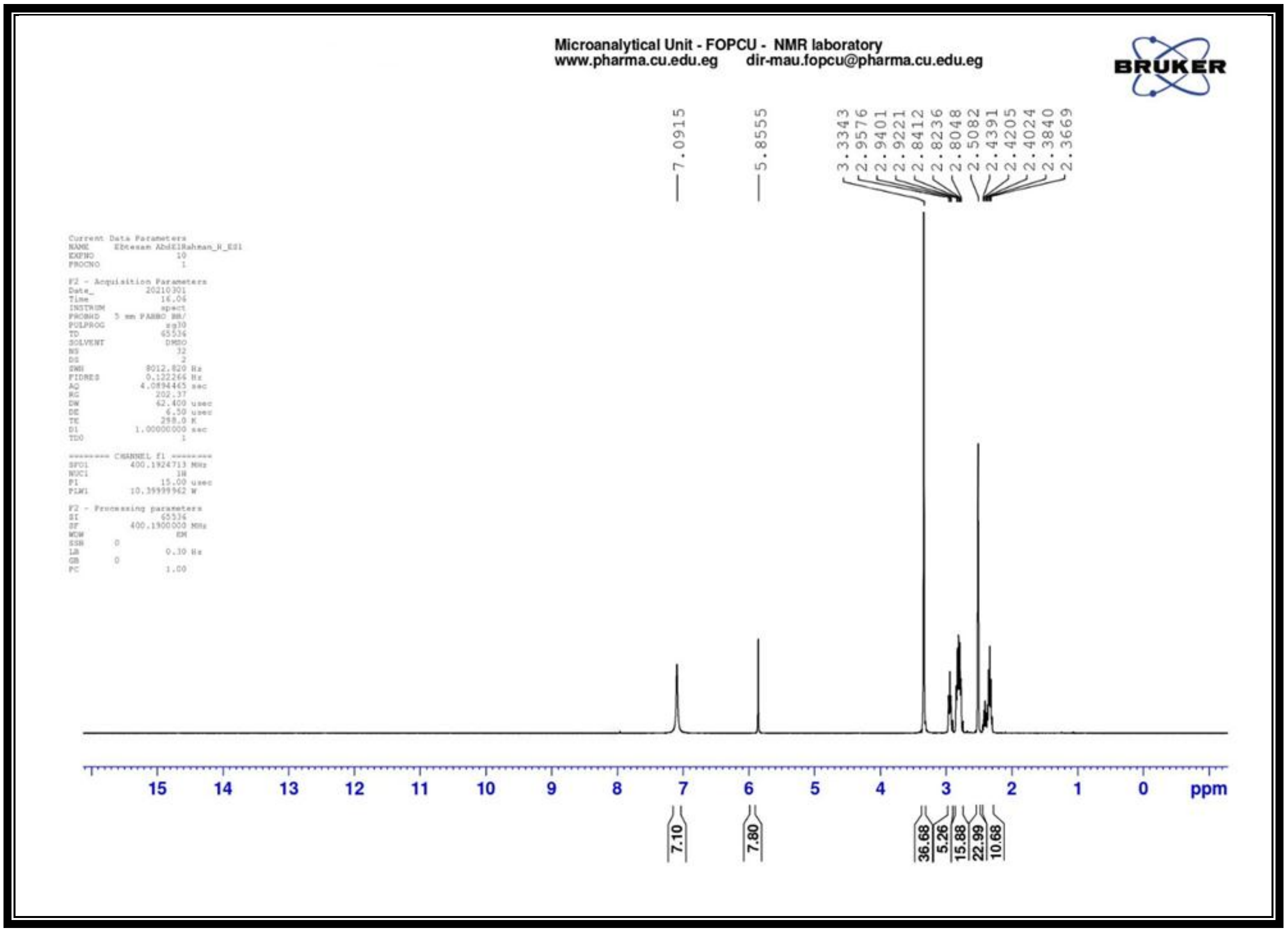

Chart S2: ^{1}H-NMR of Compound 3

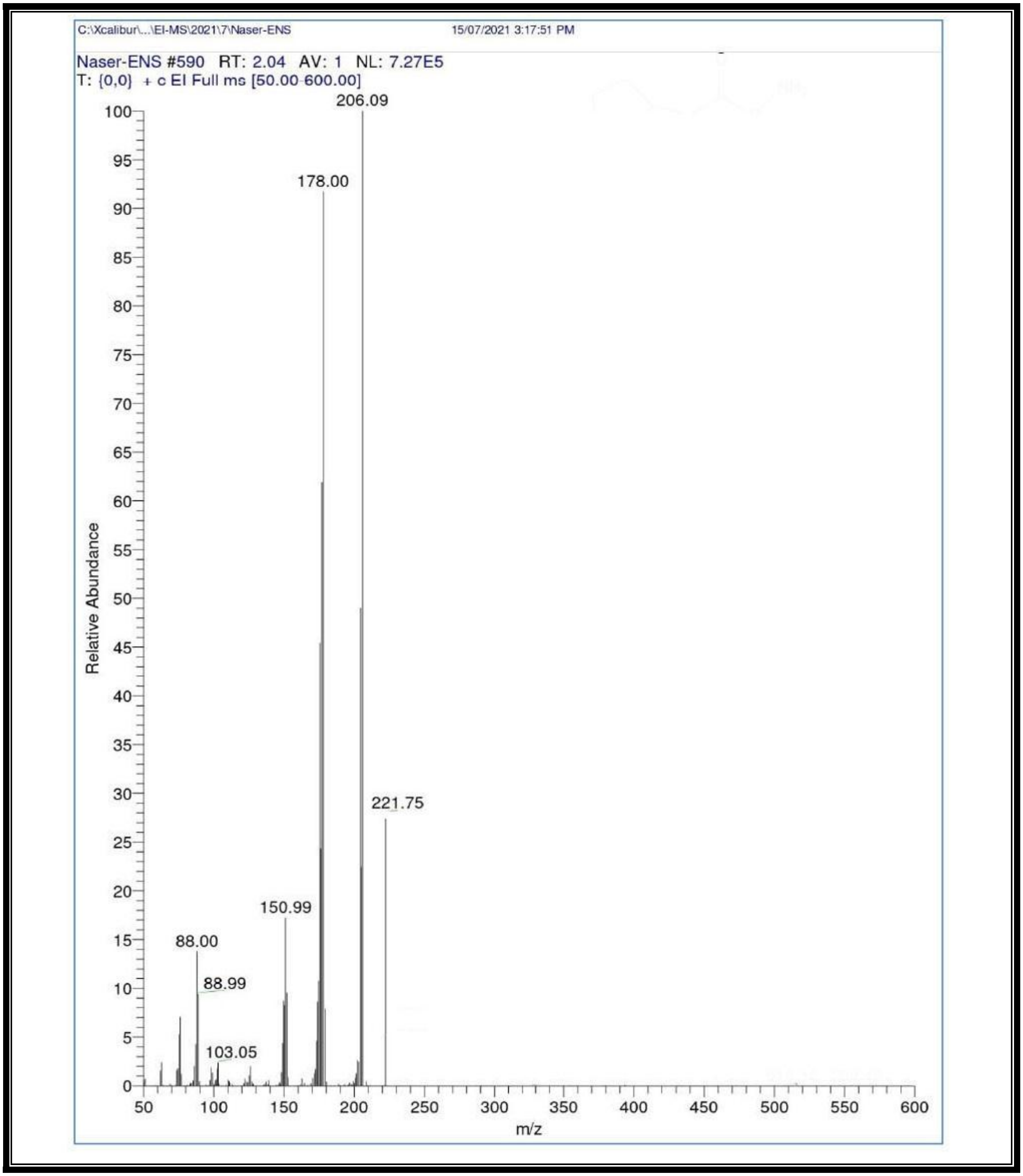

Chart S3: mass of Compound 3

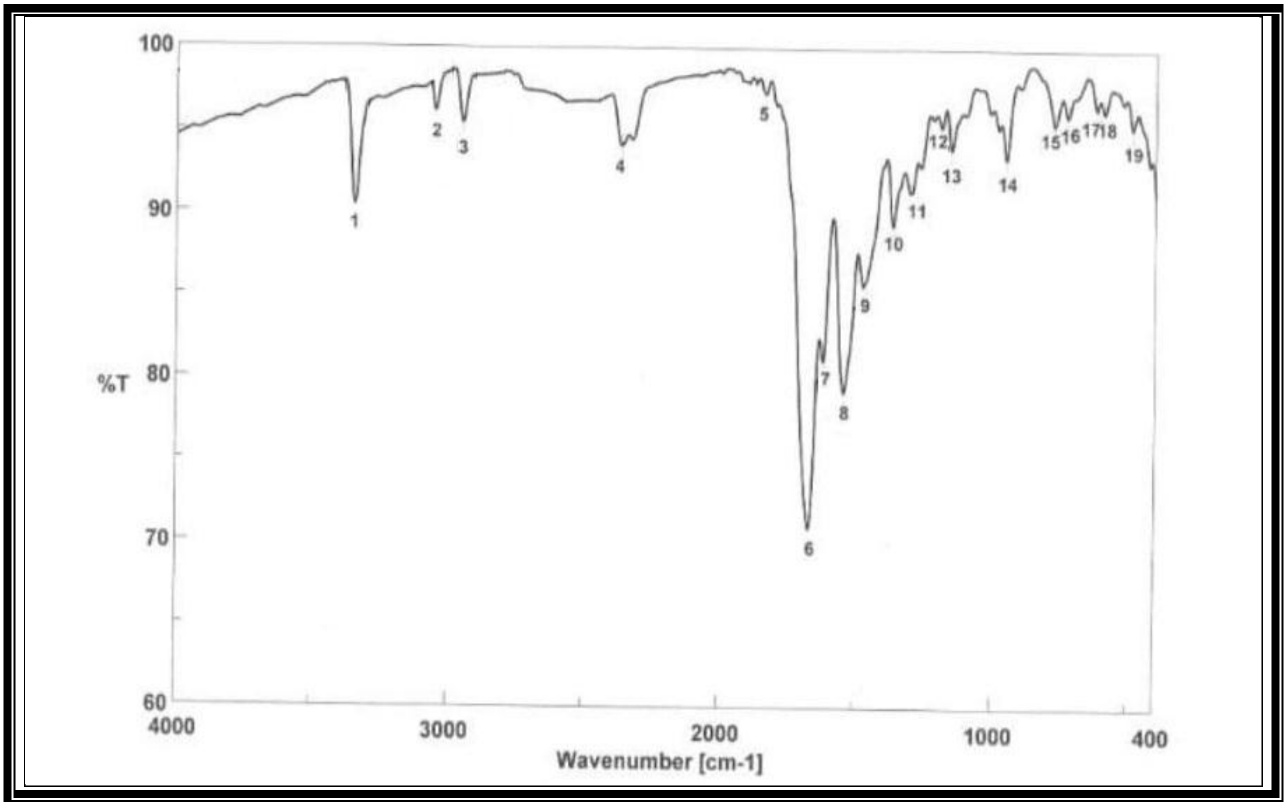

Chart S4: IR of Compound 4

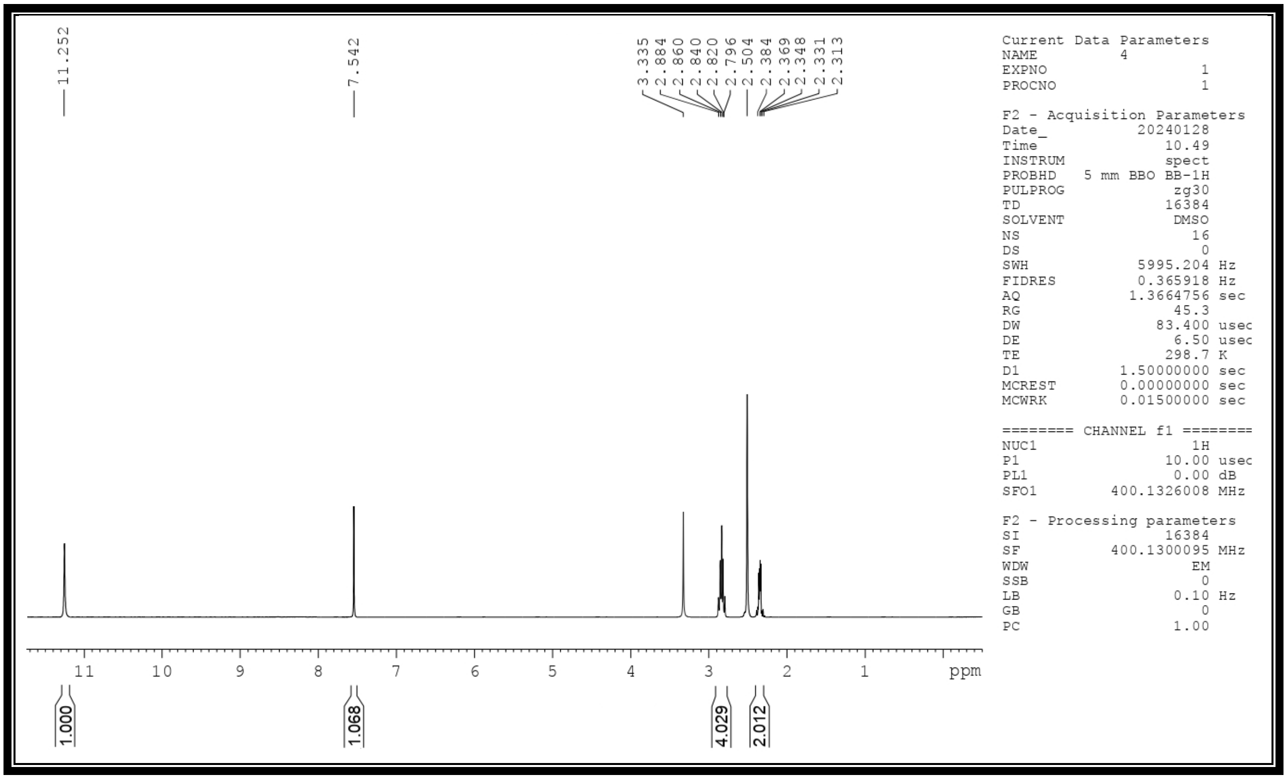

Chart S5: ^{1}H-NMR of Compound 4

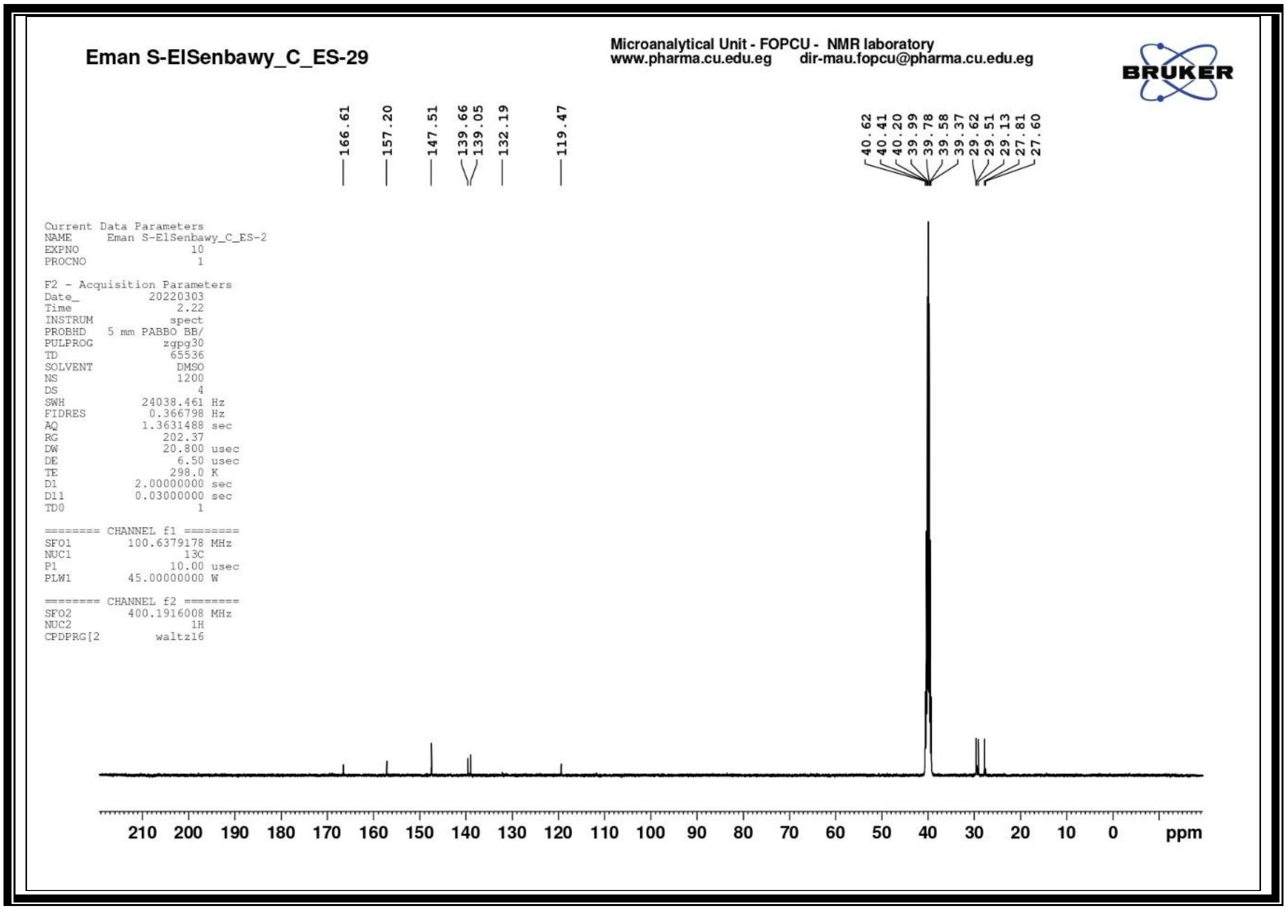

Chart S6: ^{13}C-NMR of Compound 4

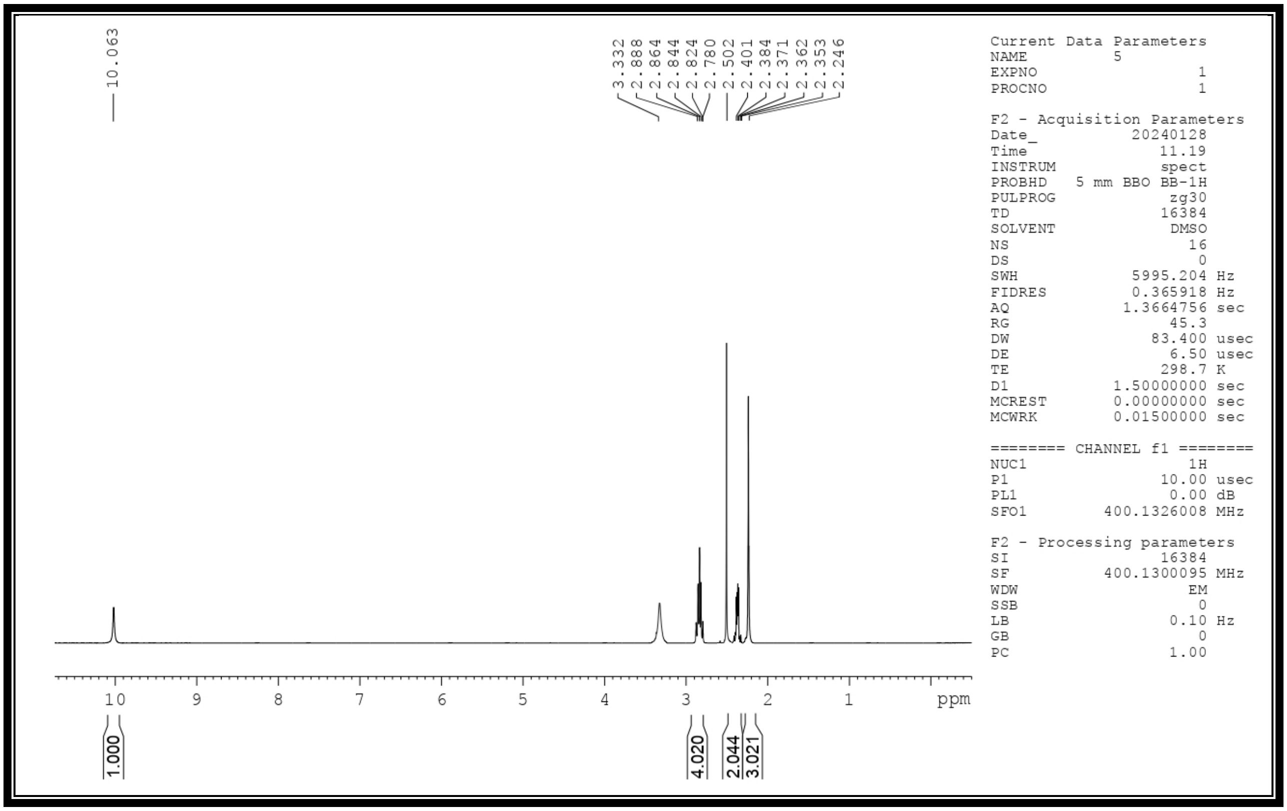

Chart S7: ^{1}H-NMR of Compound 5

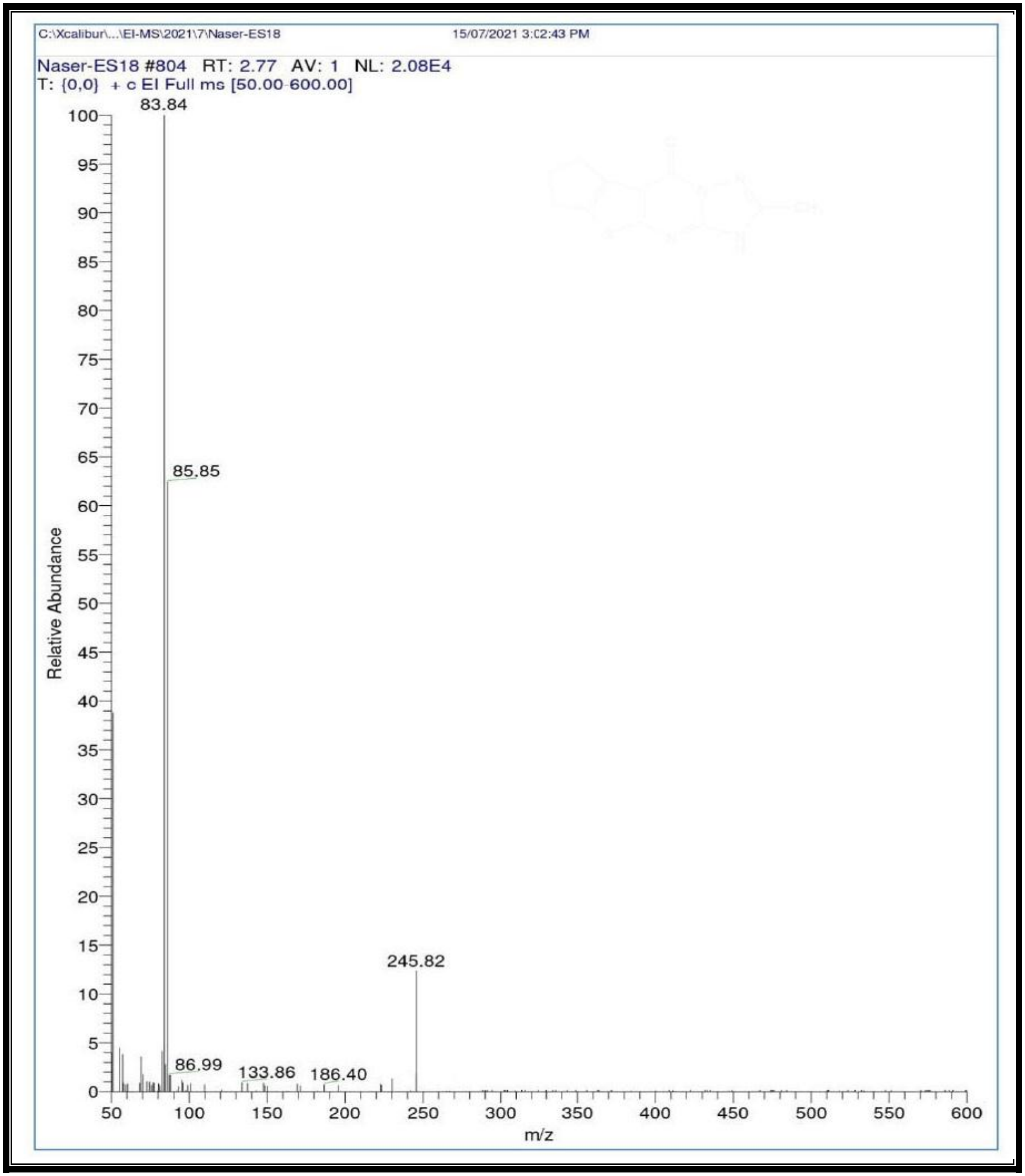

Chart S8: mass of Compound 5

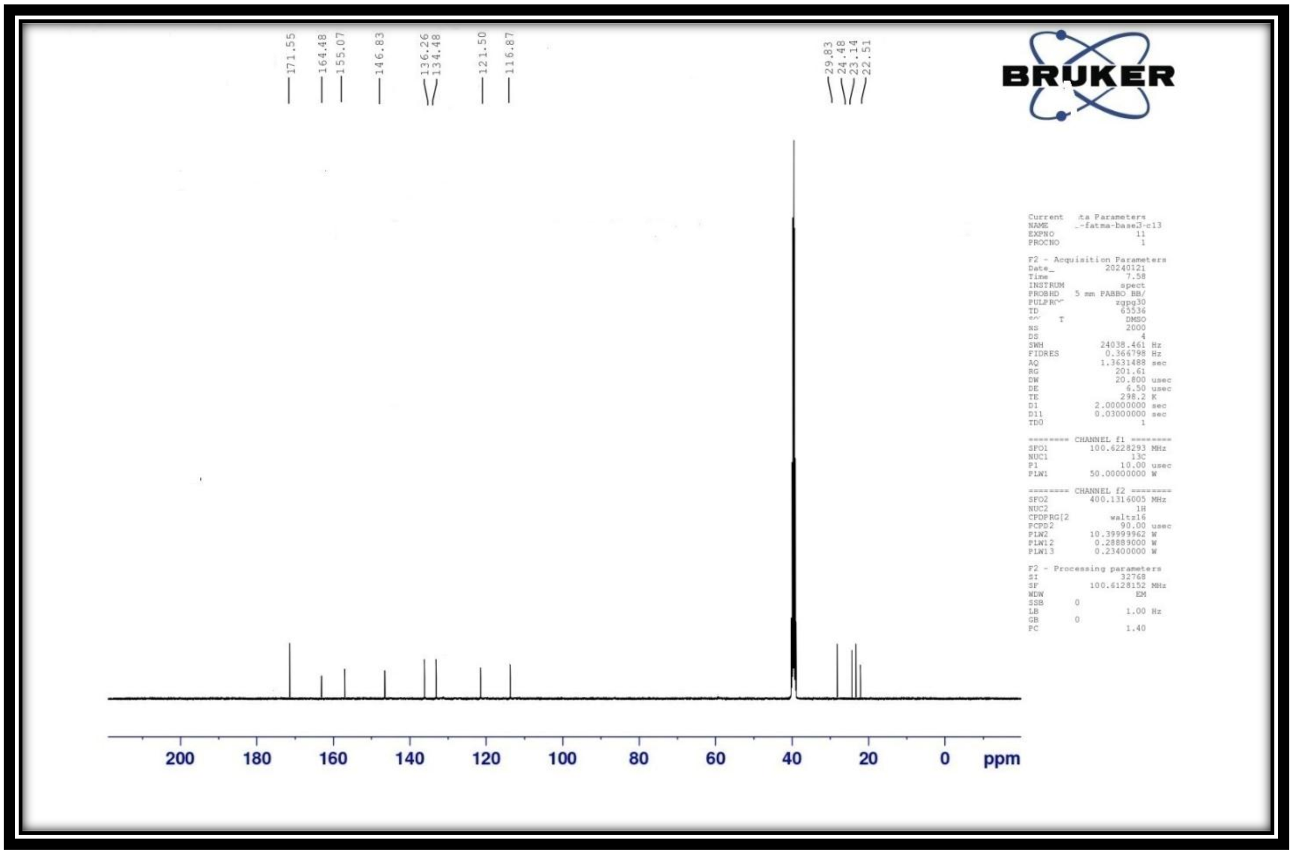

Chart S9: ^{13}C-NMR of Compound 7

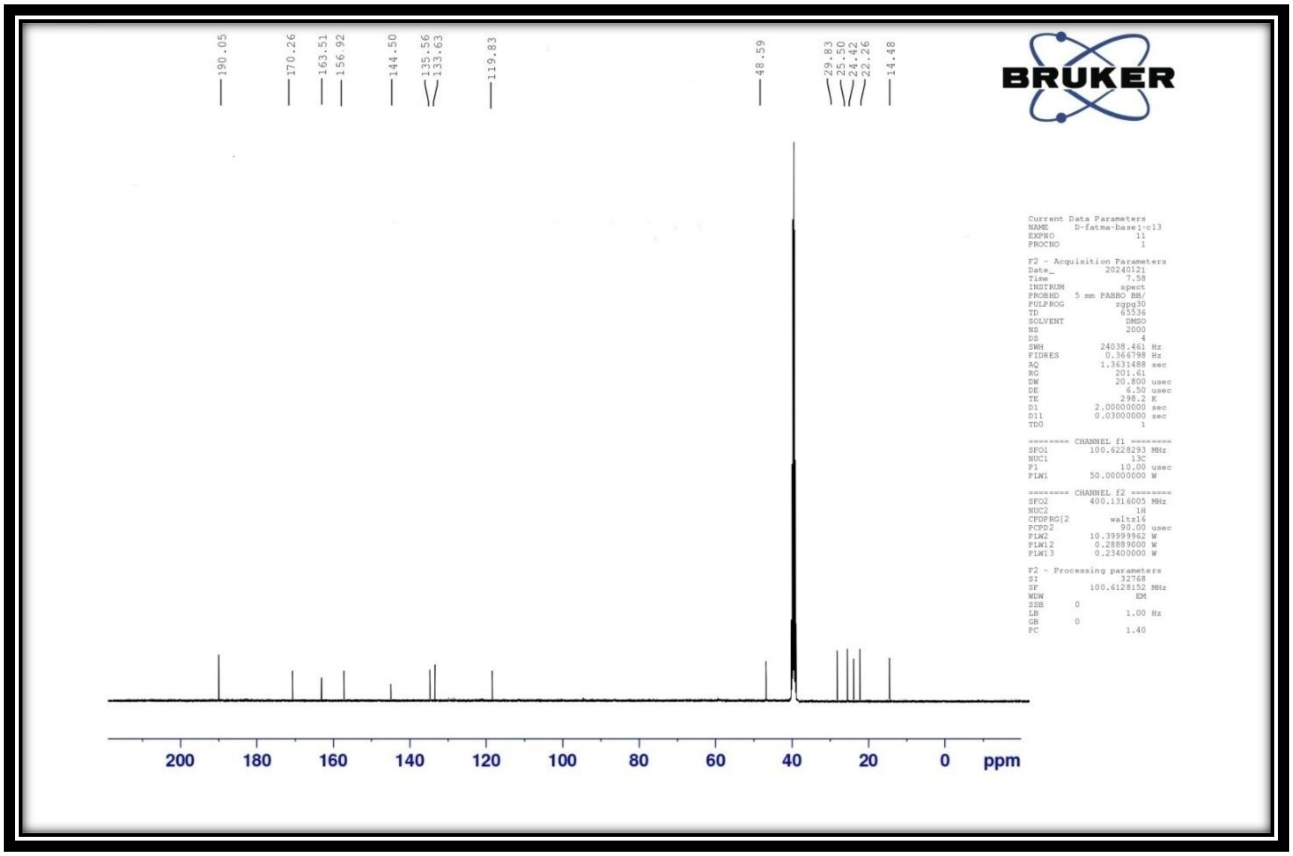

Chart S10: ^{13}C-NMR of Compound 8

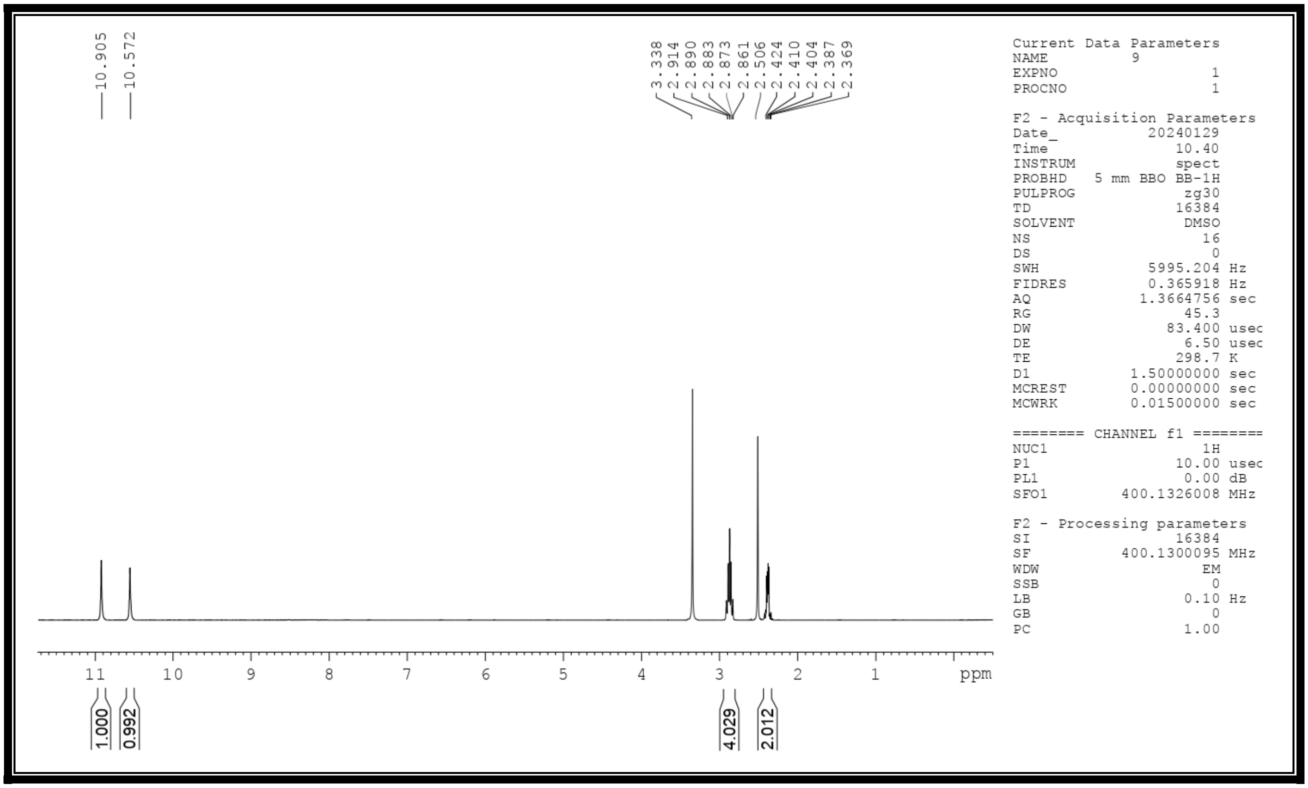

Chart S11: ^{1}H-NMR of Compound 9

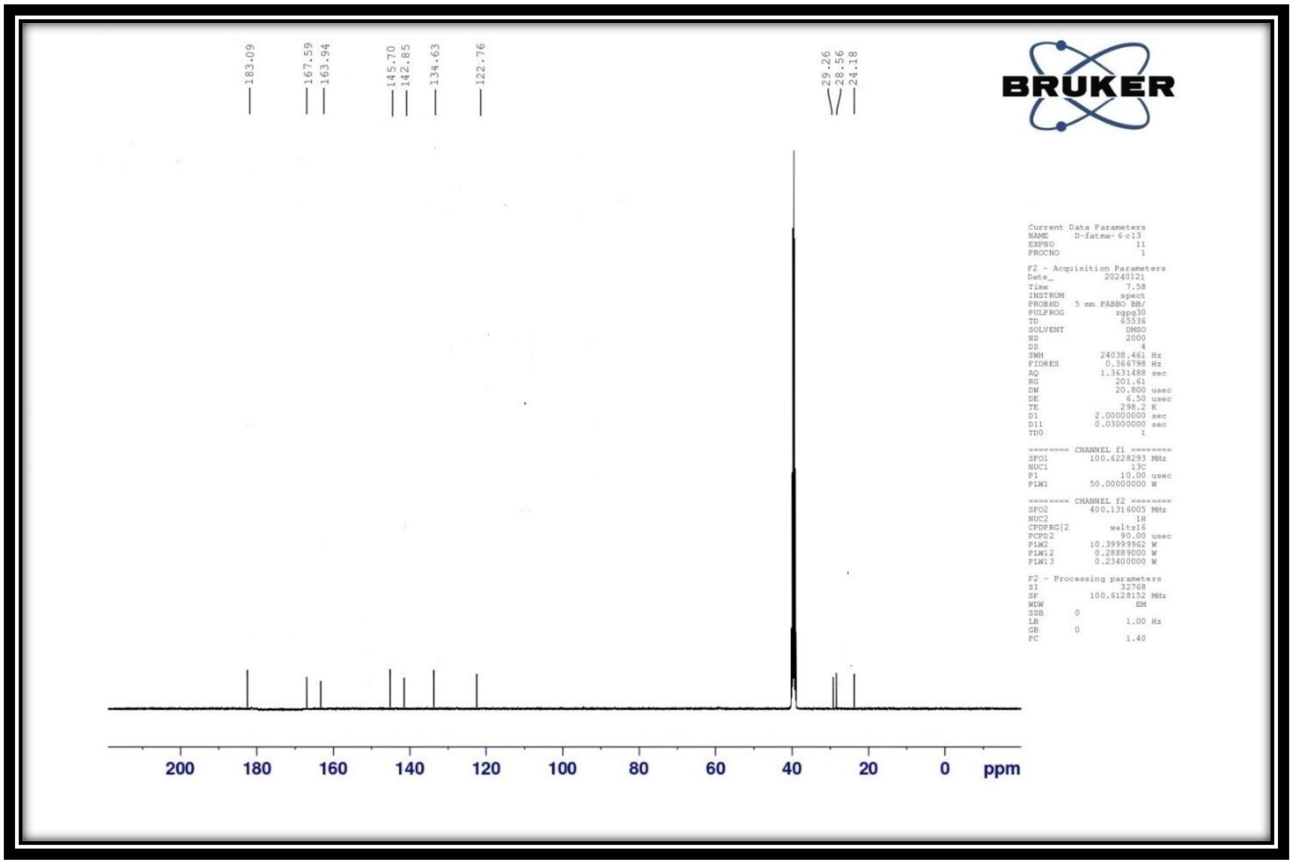

Chart S12: ^{13}C-NMR of Compound 9

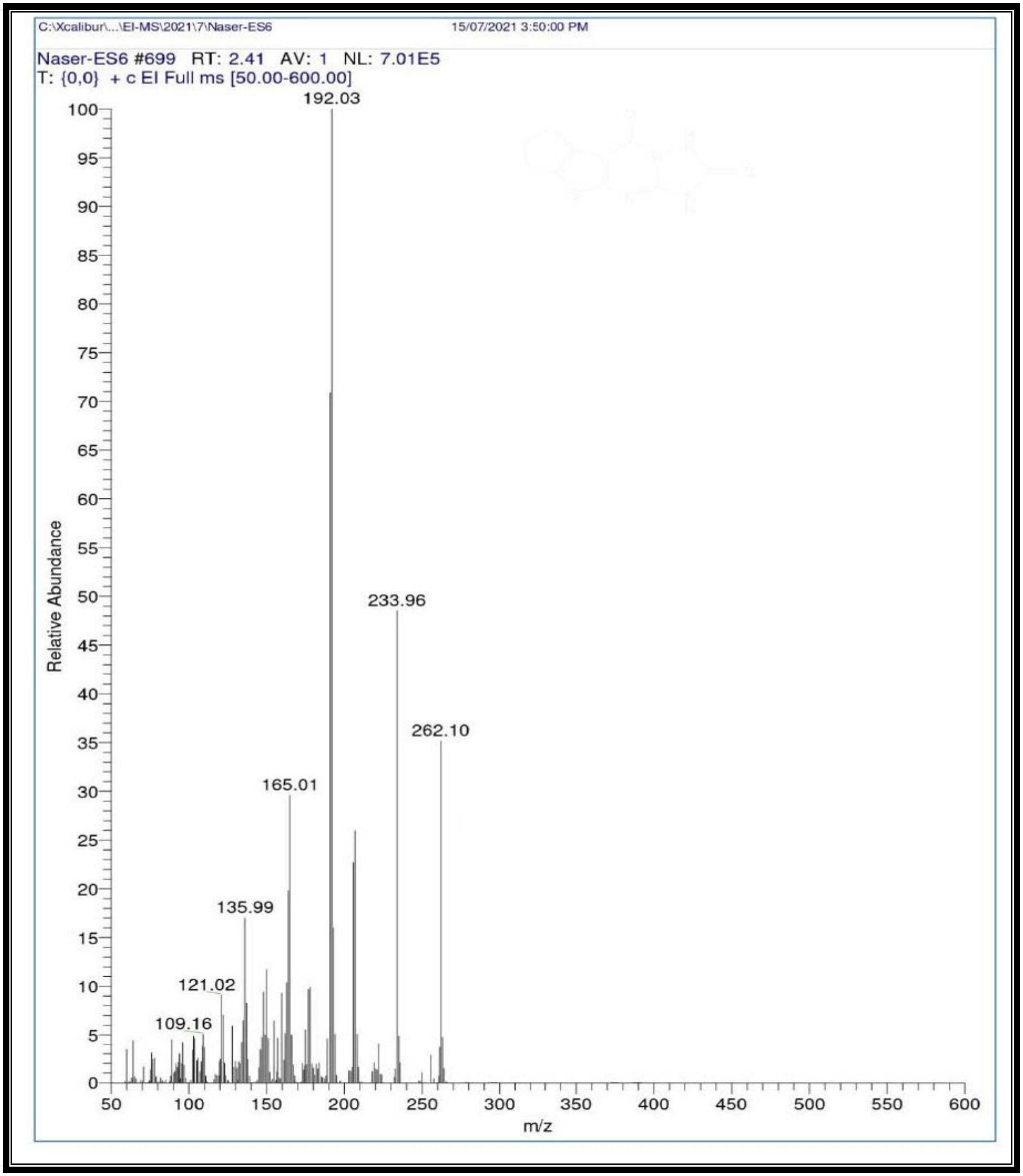

Chart S13: mass of Compound 9

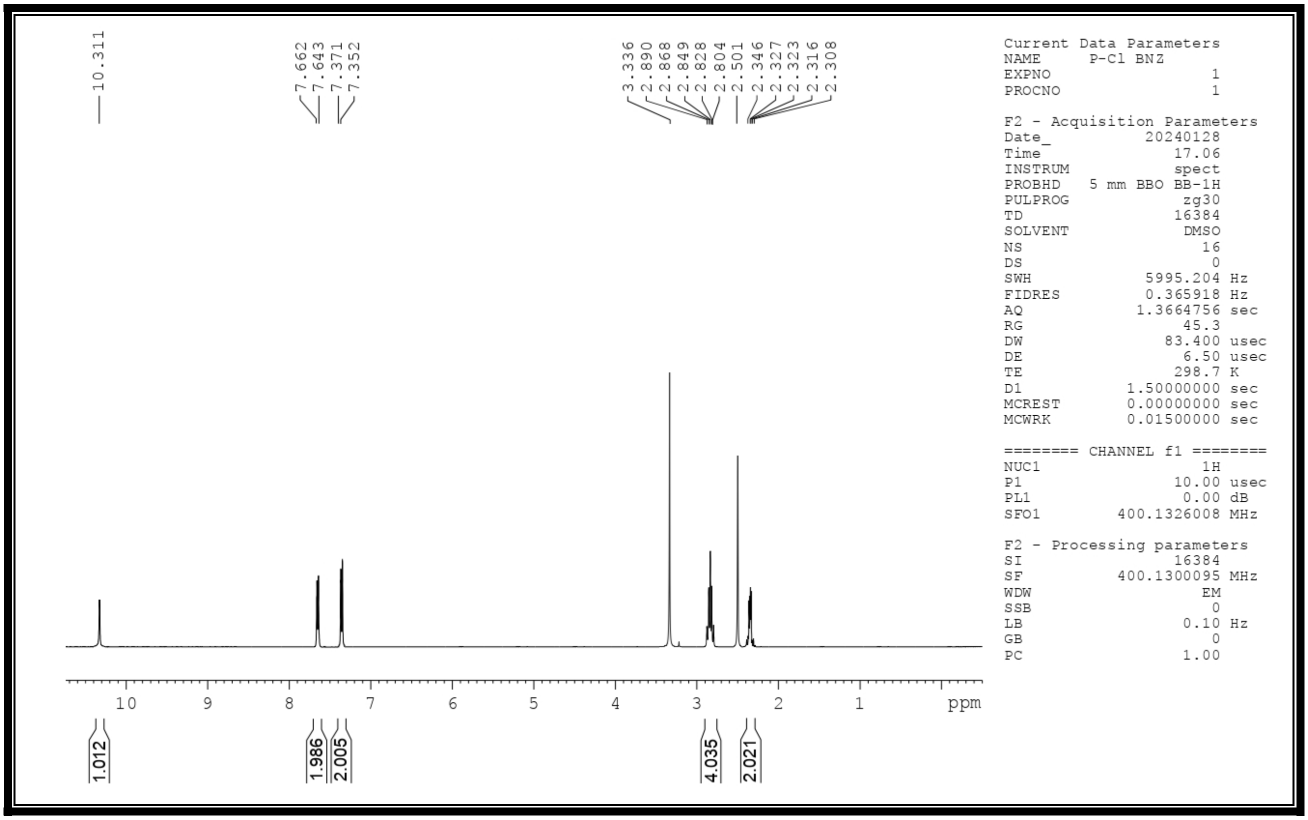

Chart S14: ^{1}H-NMR of Compound 10a

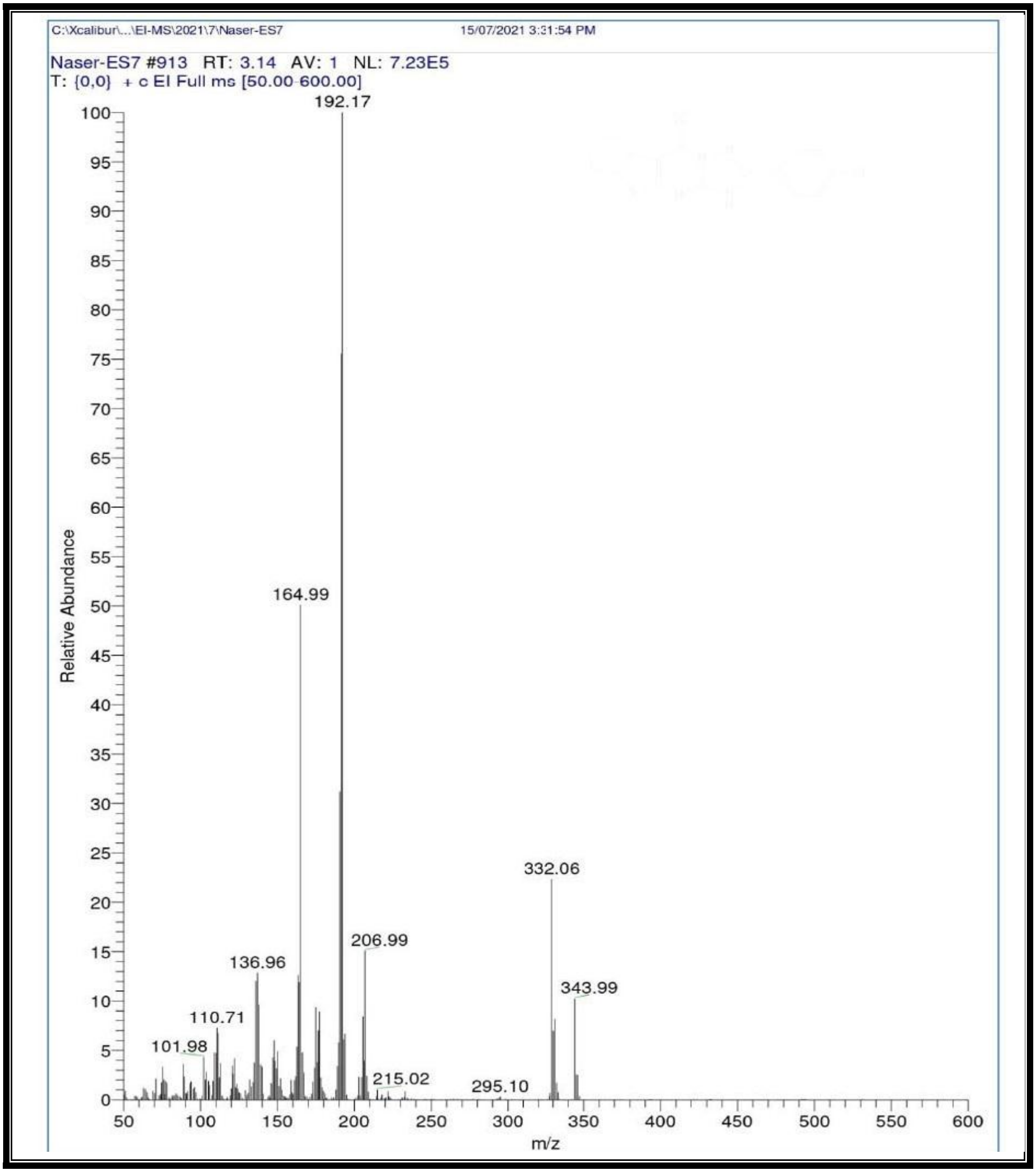

Chart S15: Mass of Compound 10a

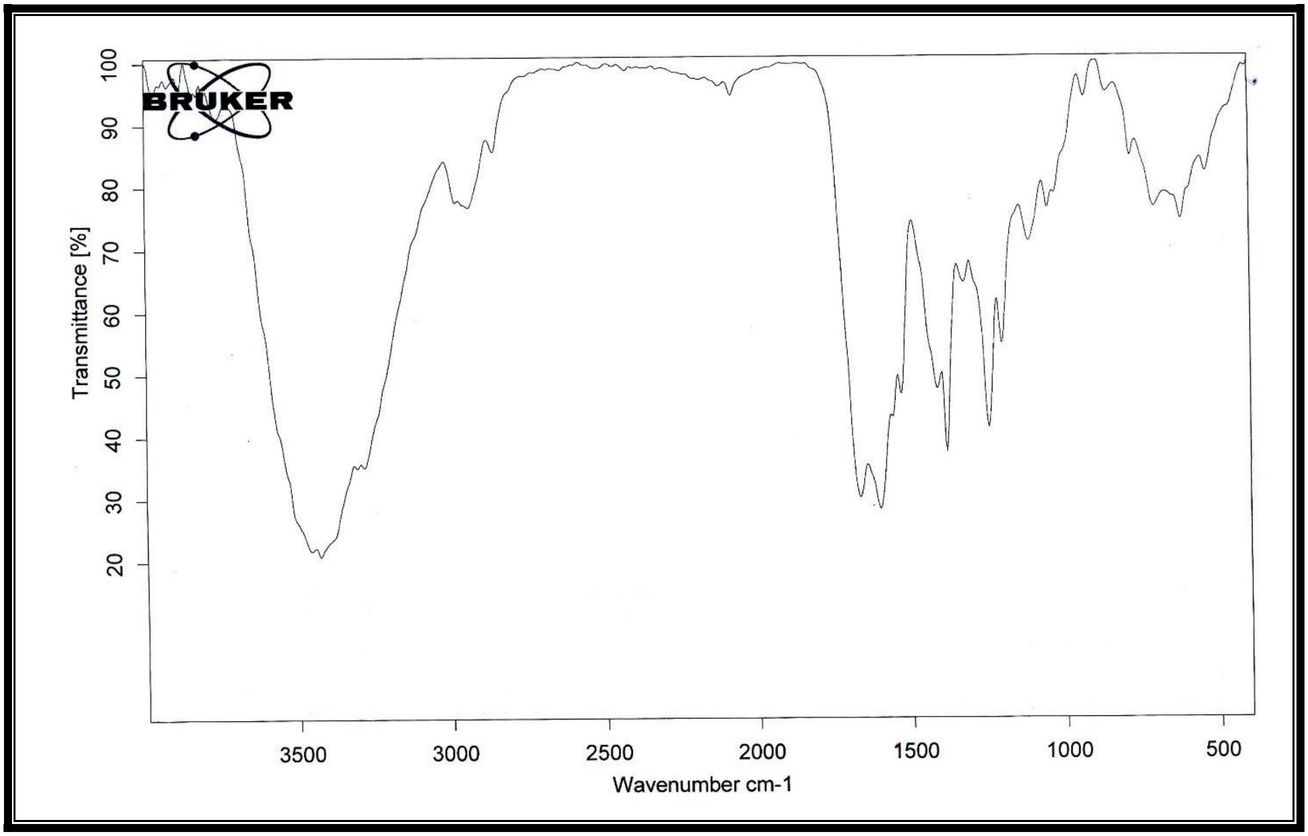

Chart S16: IR of Compound 10b

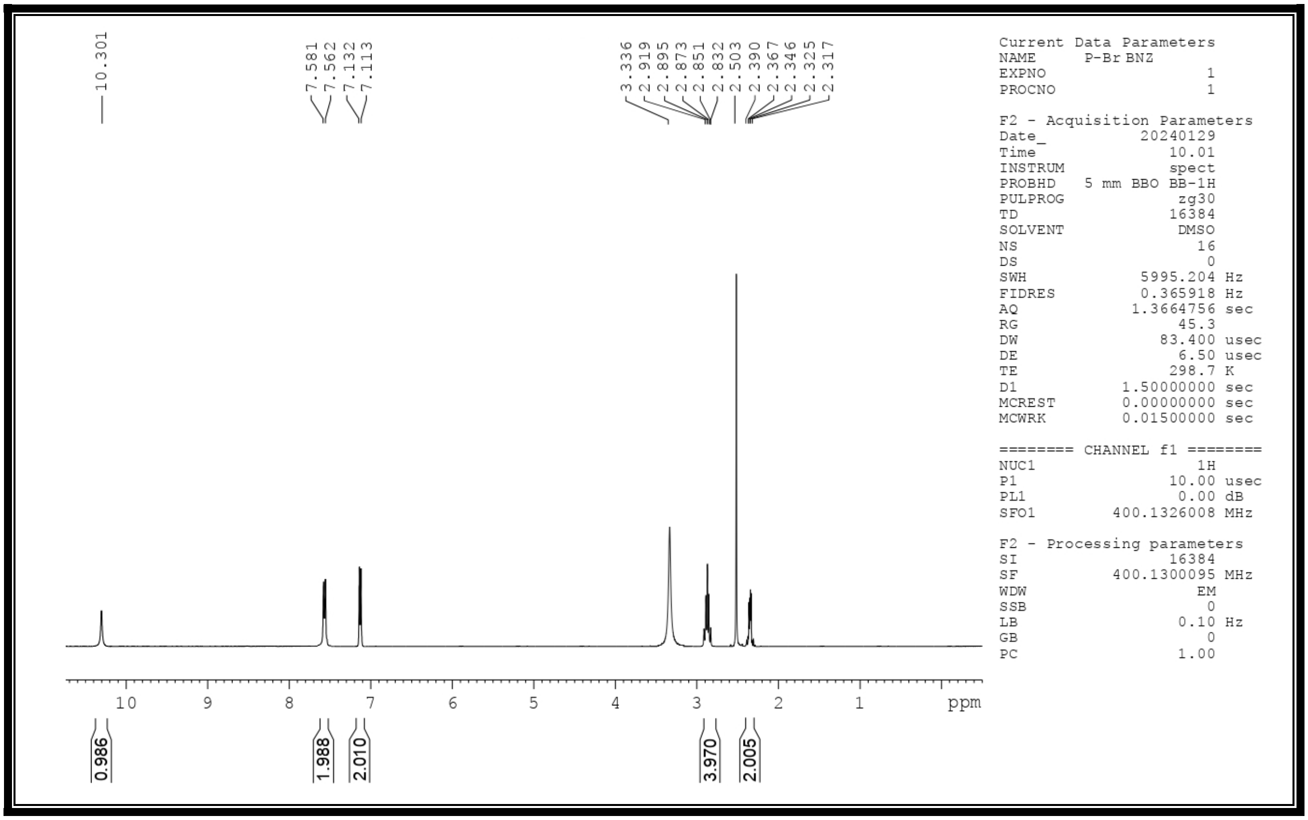

Chart S17: 1H-NMR of Compound 10b

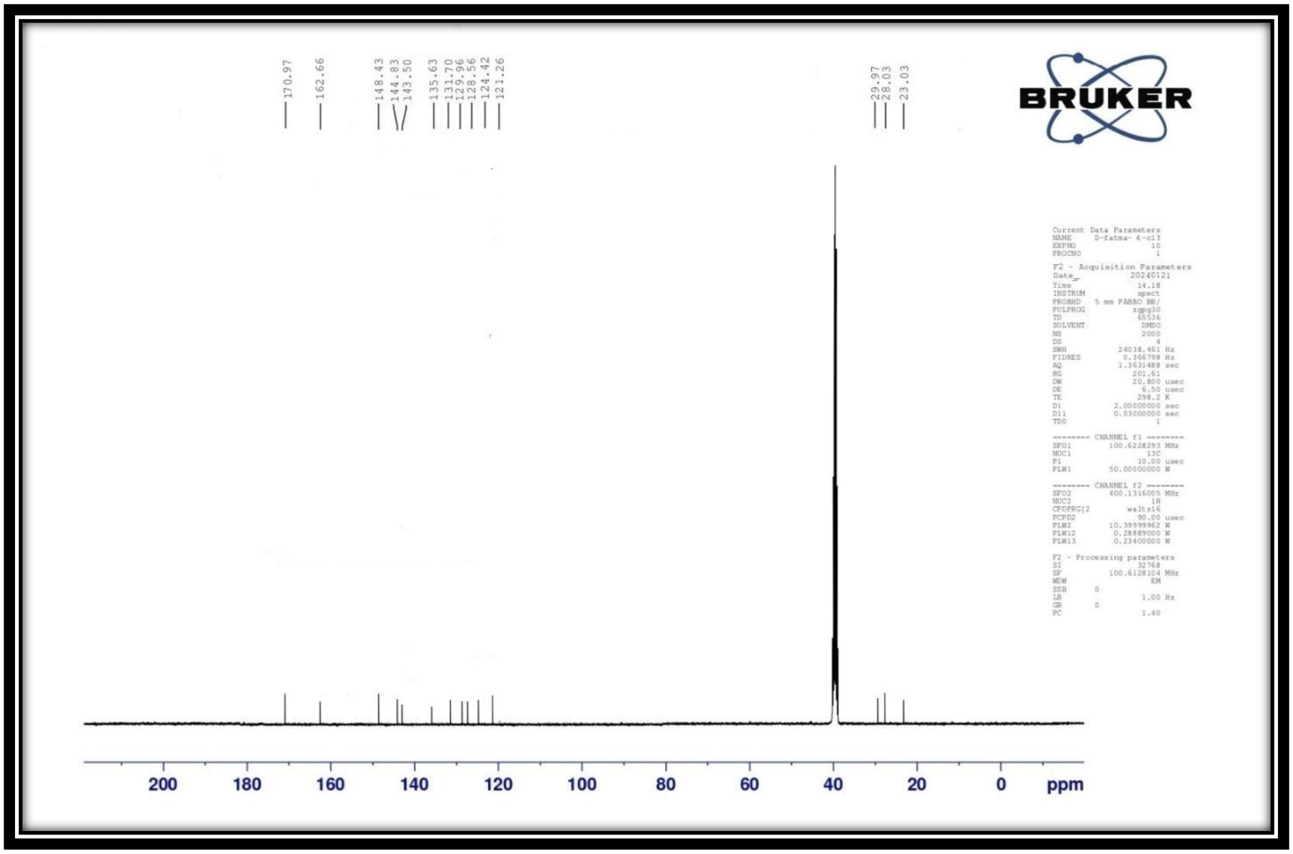

Chart S18: ^{13}C-NMR of Compound 10b

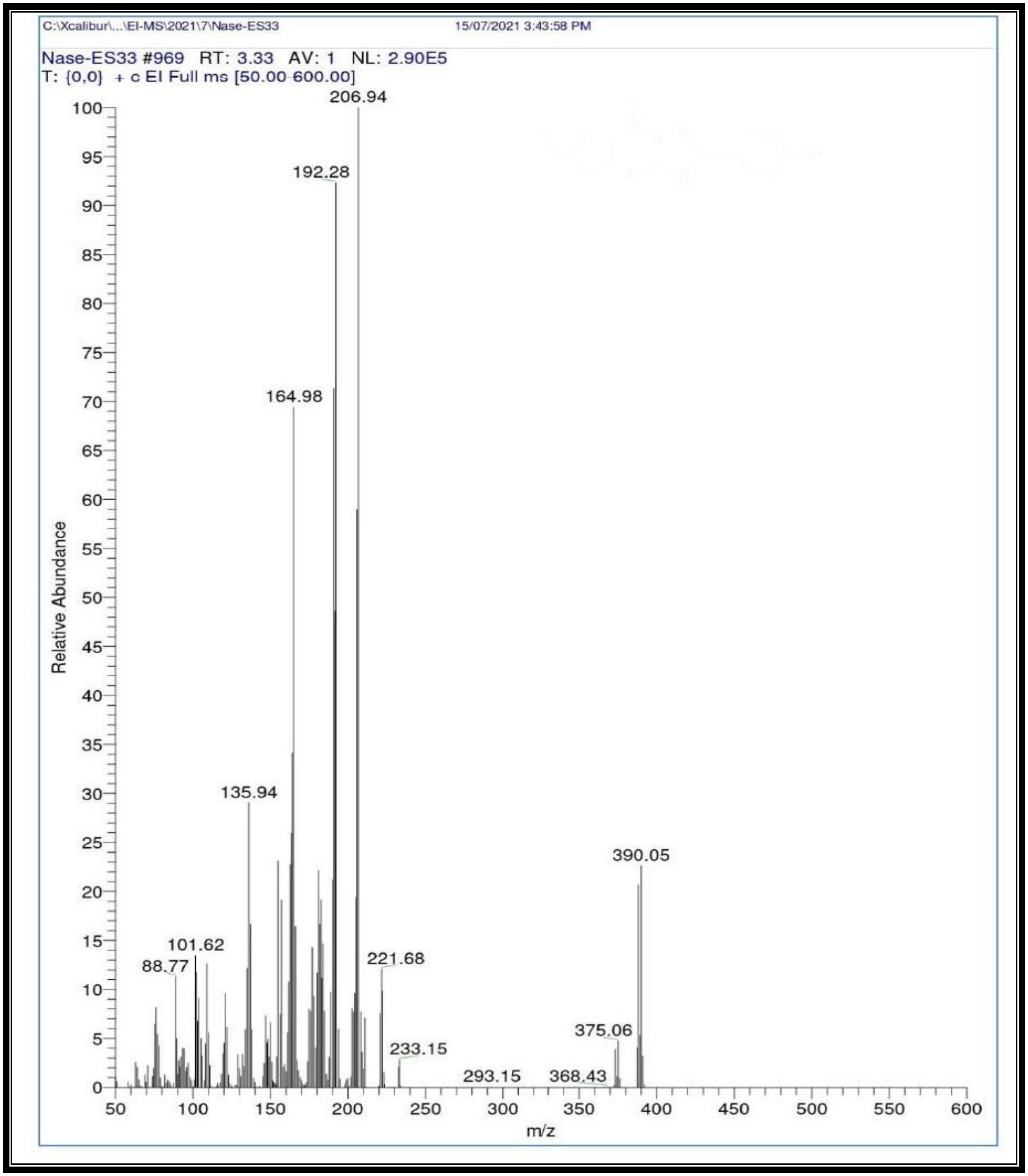

Chart S19: Mass of Compound 10b

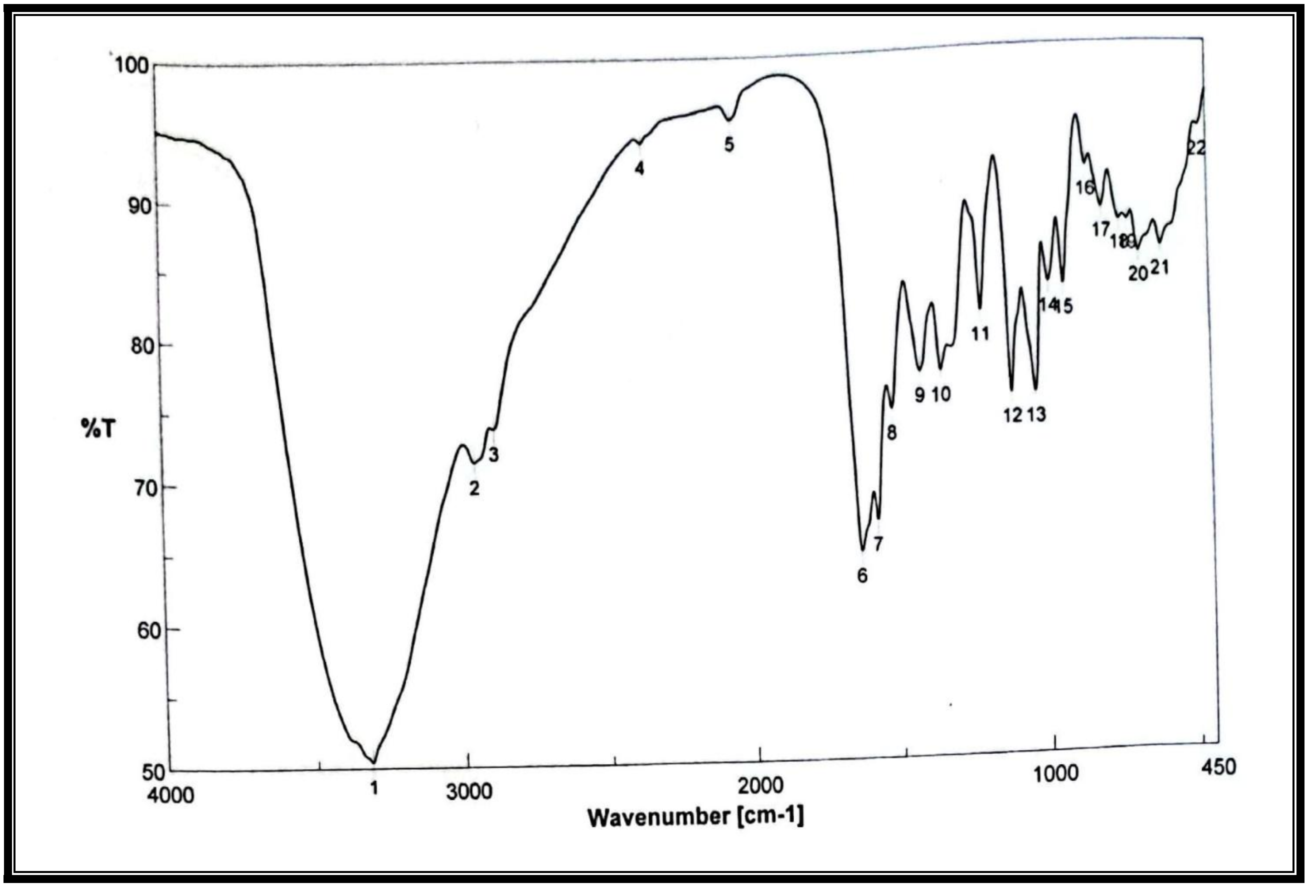

Chart S20: IR of Compound 10c

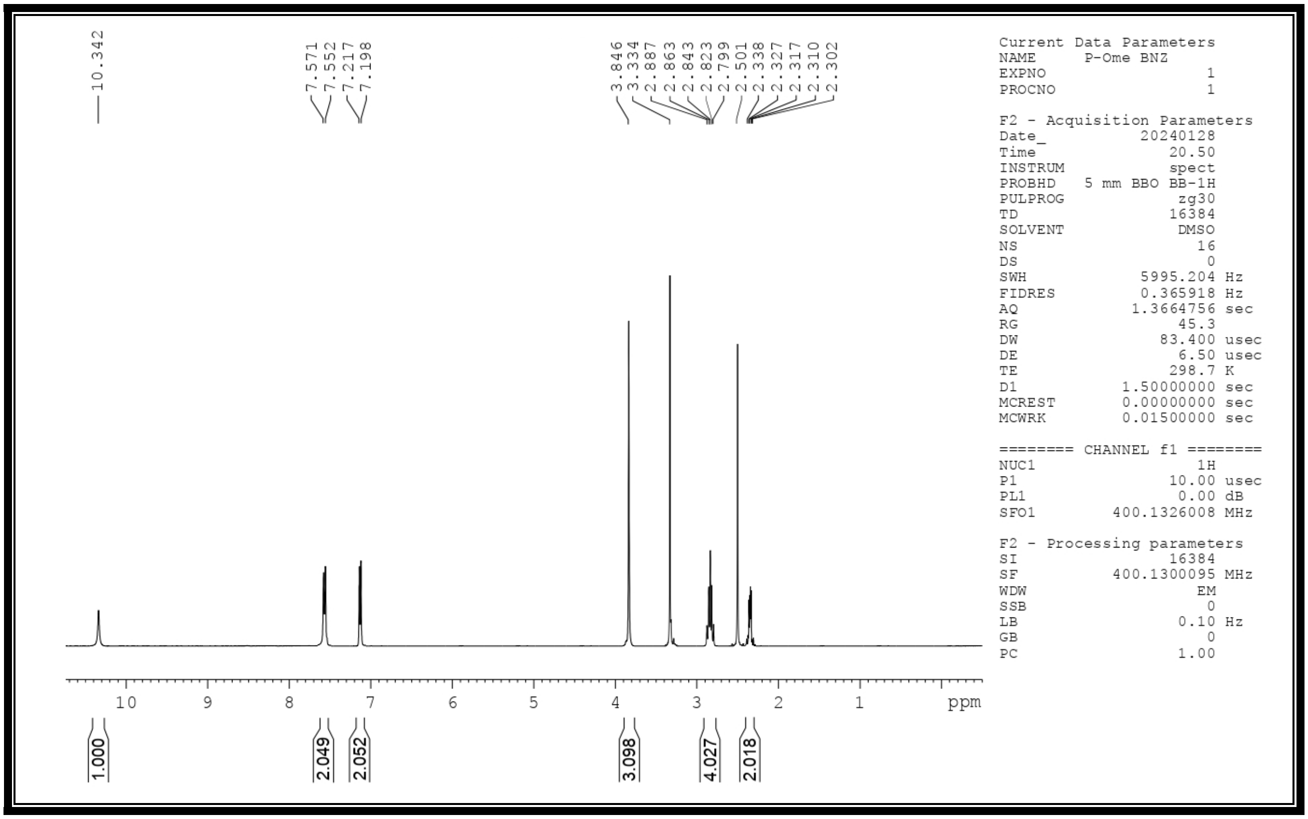

Chart S21: ^{1}H-NMR of Compound 10d

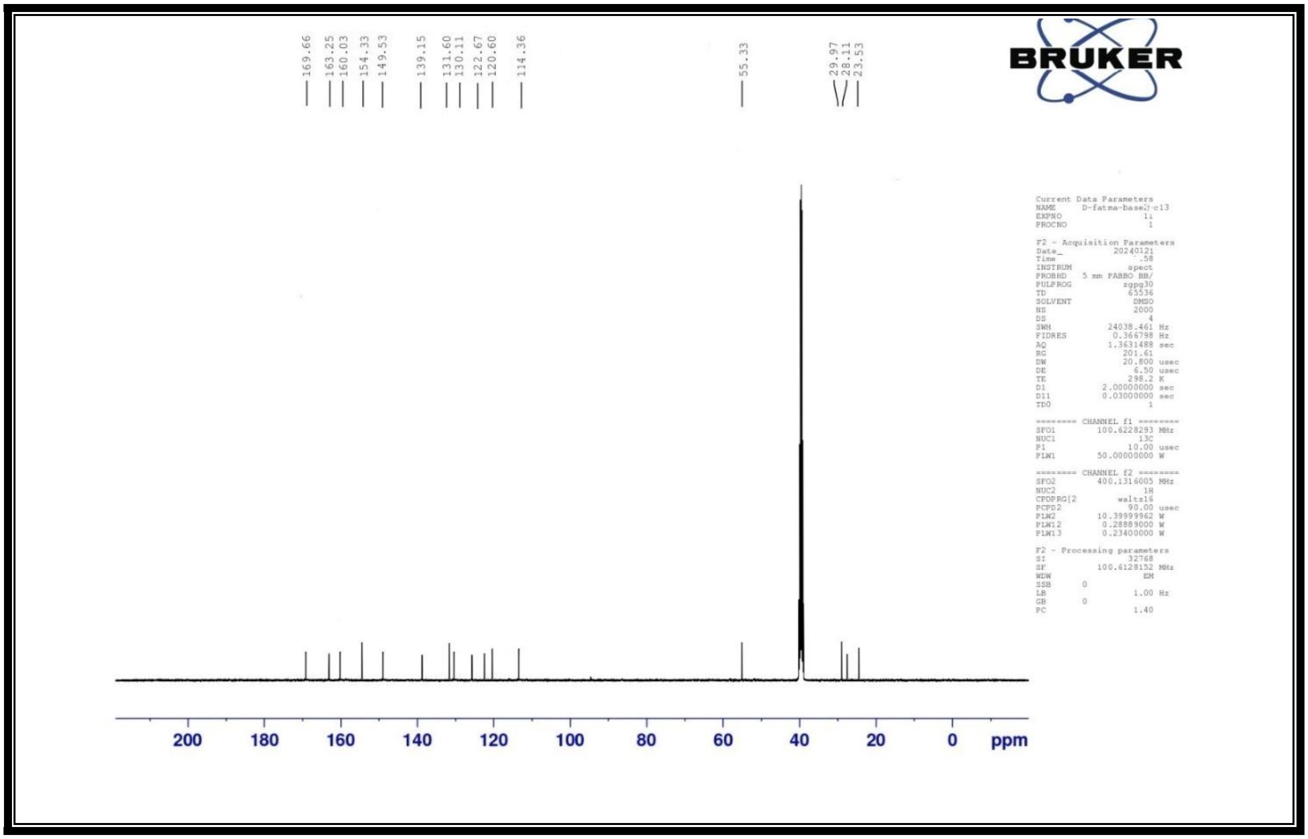

Chart S22: ^{13}C-NMR of Compound 10d

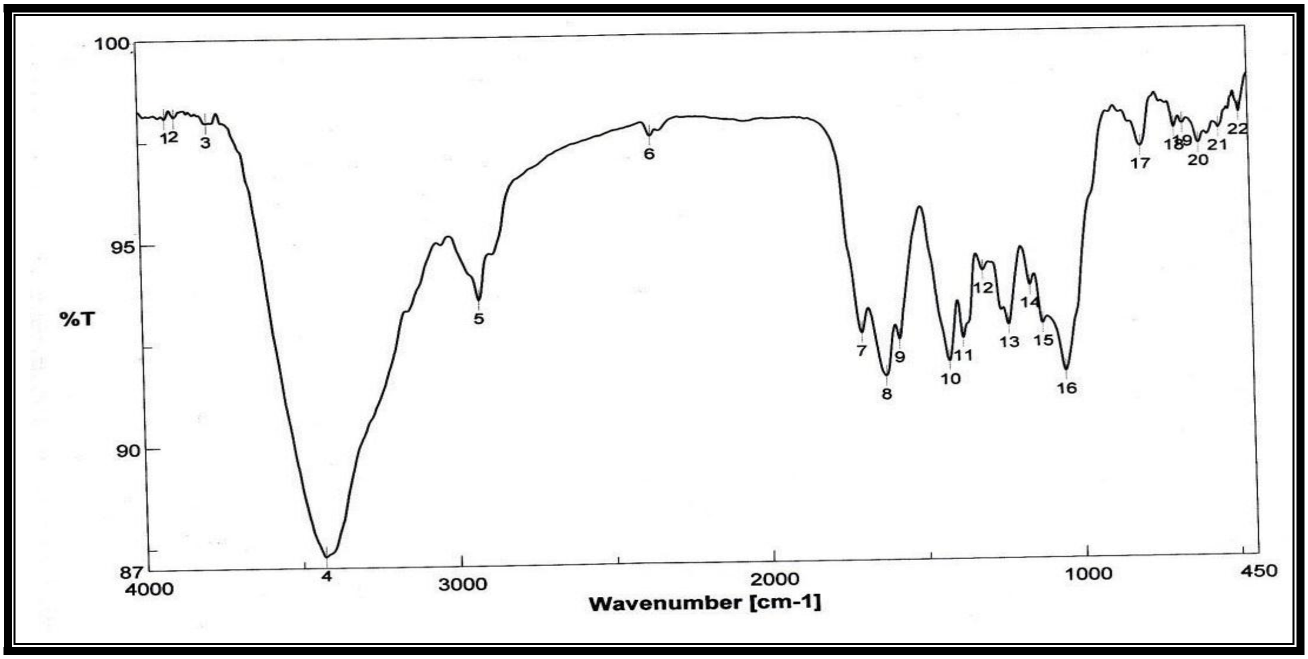

Chart S23: IR of Compound 11a

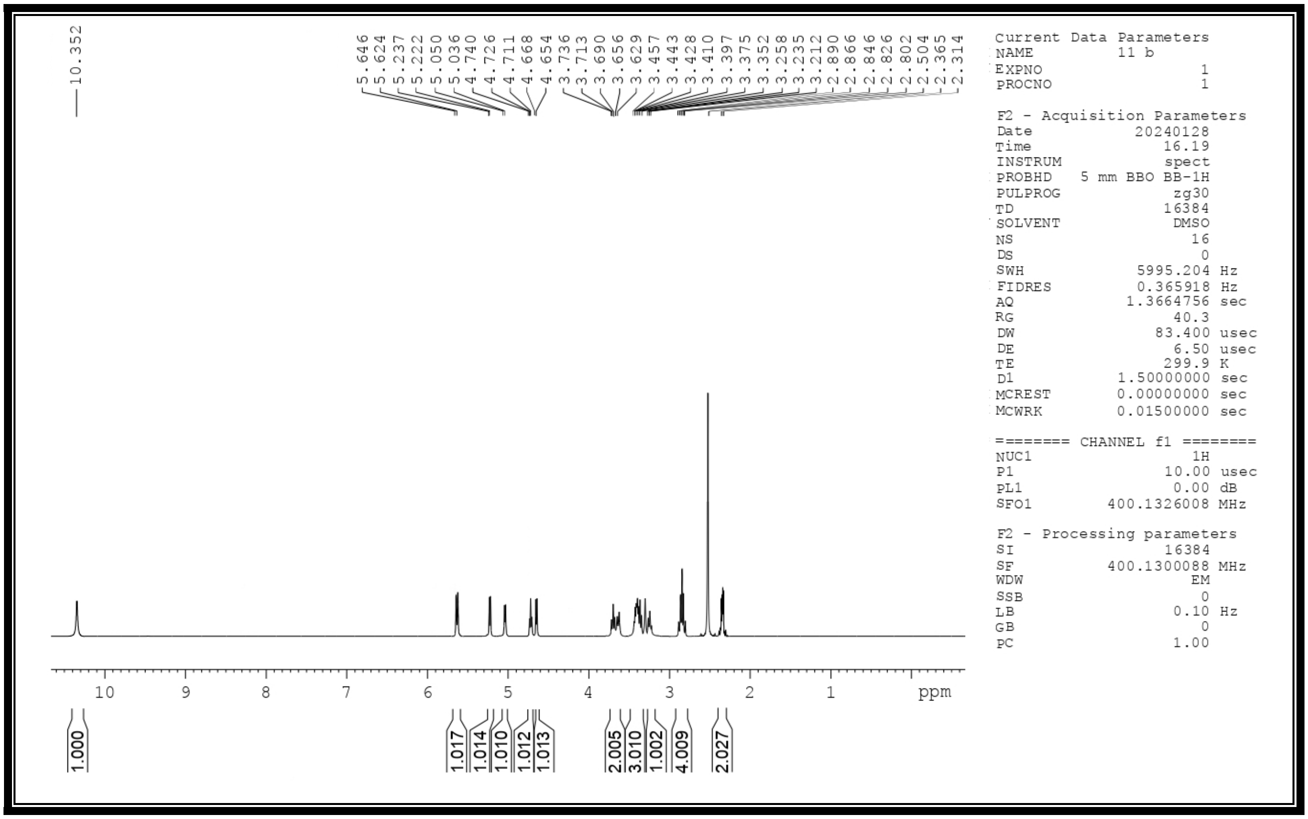

Chart S24: ^{1}H-NMR of Compound 11b

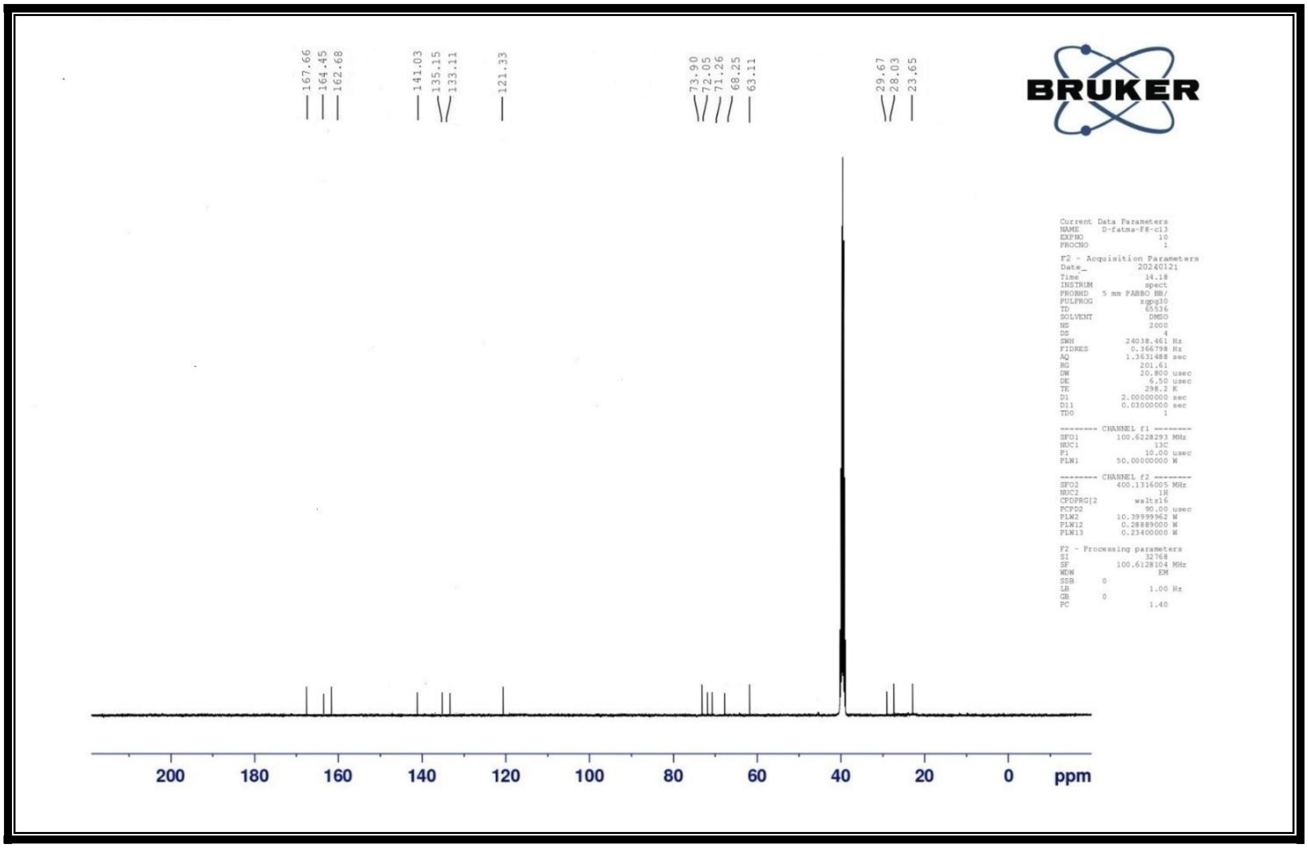

Chart S25: ^{13}C-NMR of Compound 11b

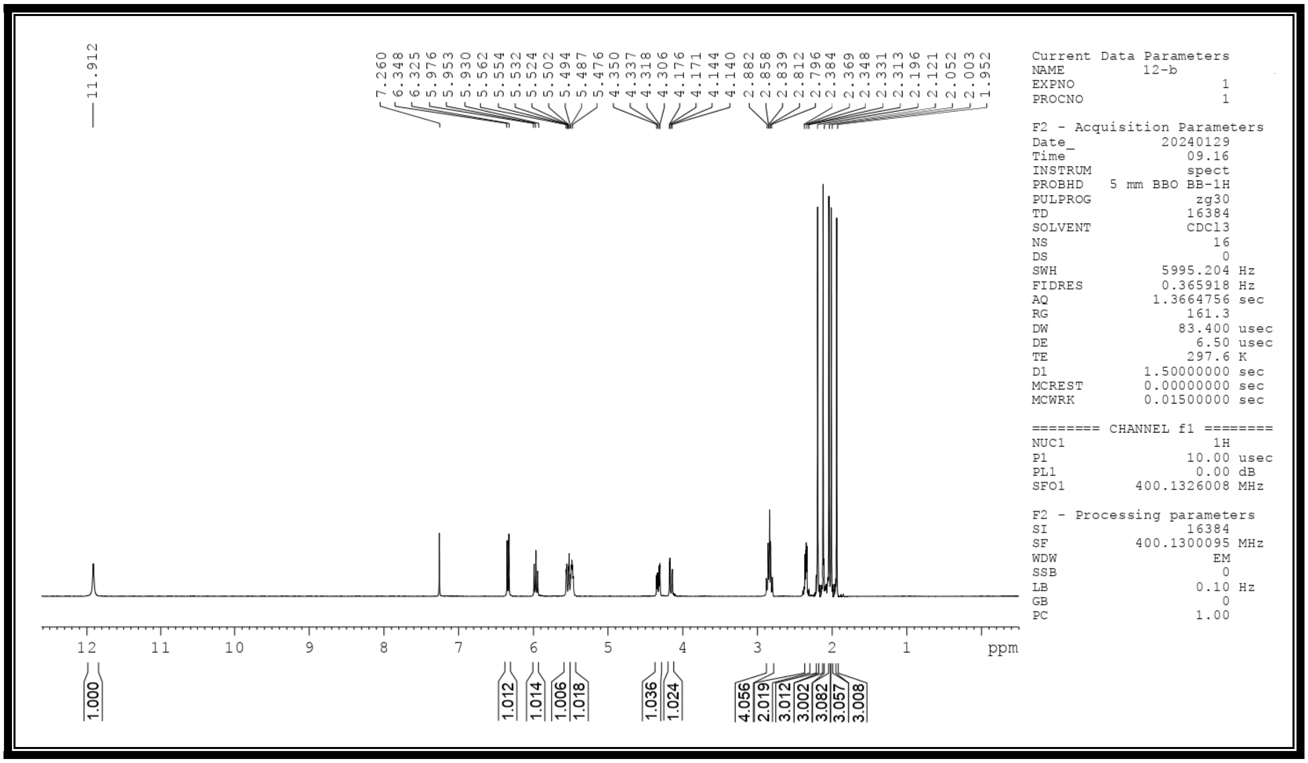

Chart S26: ^{1}H-NMR of Compound 12b

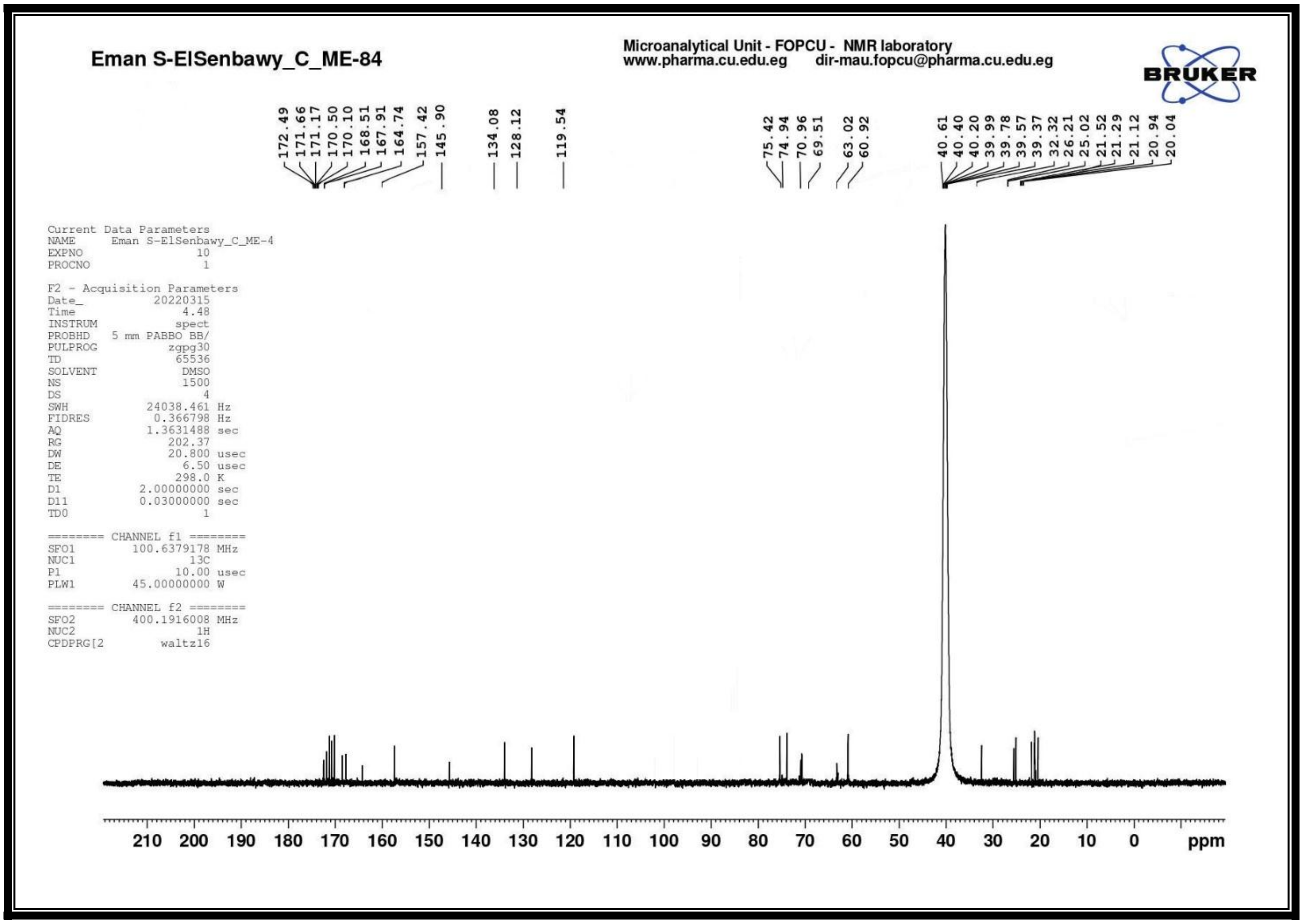

Chart S27: ^{13}C-NMR of Compound 12b

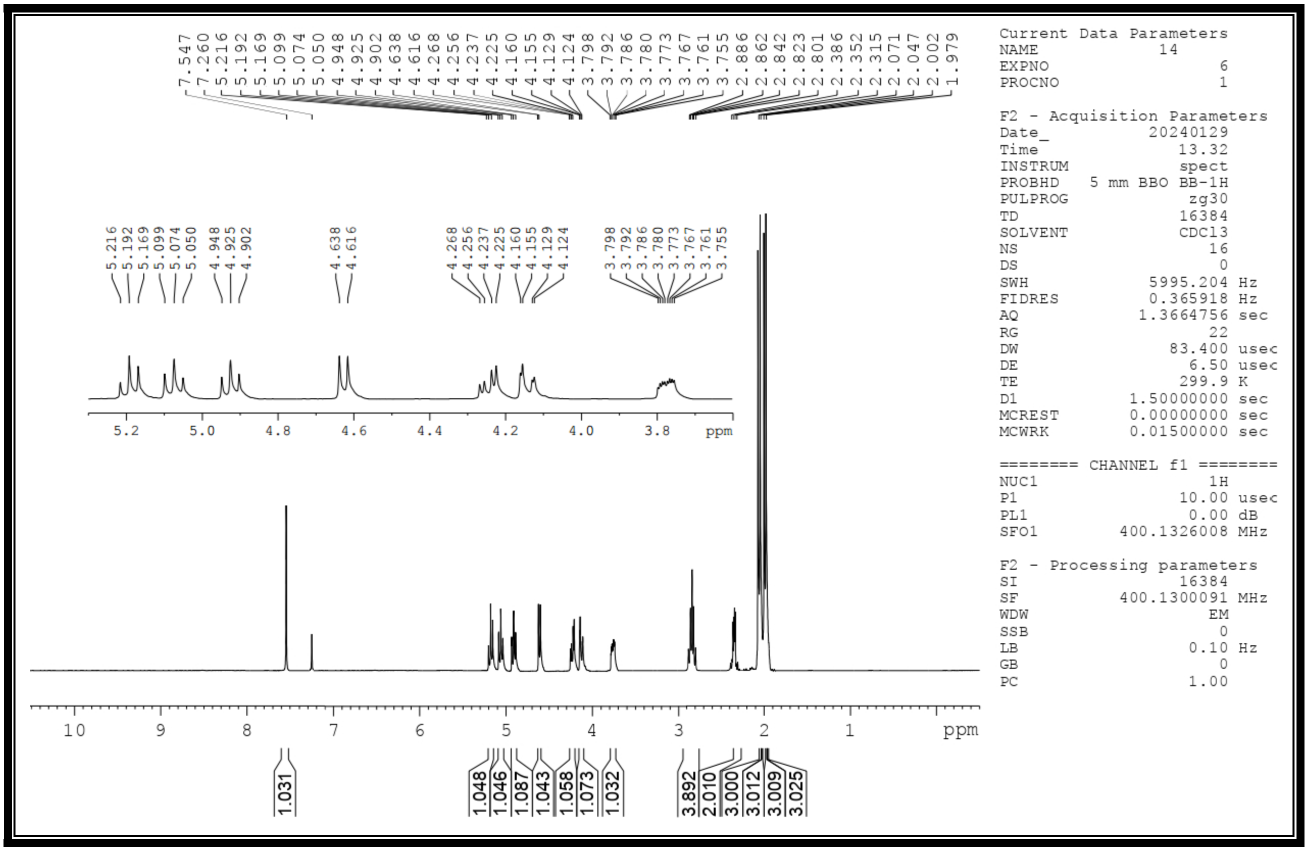

Chart S28: ^{1}H-NMR of Compound 14

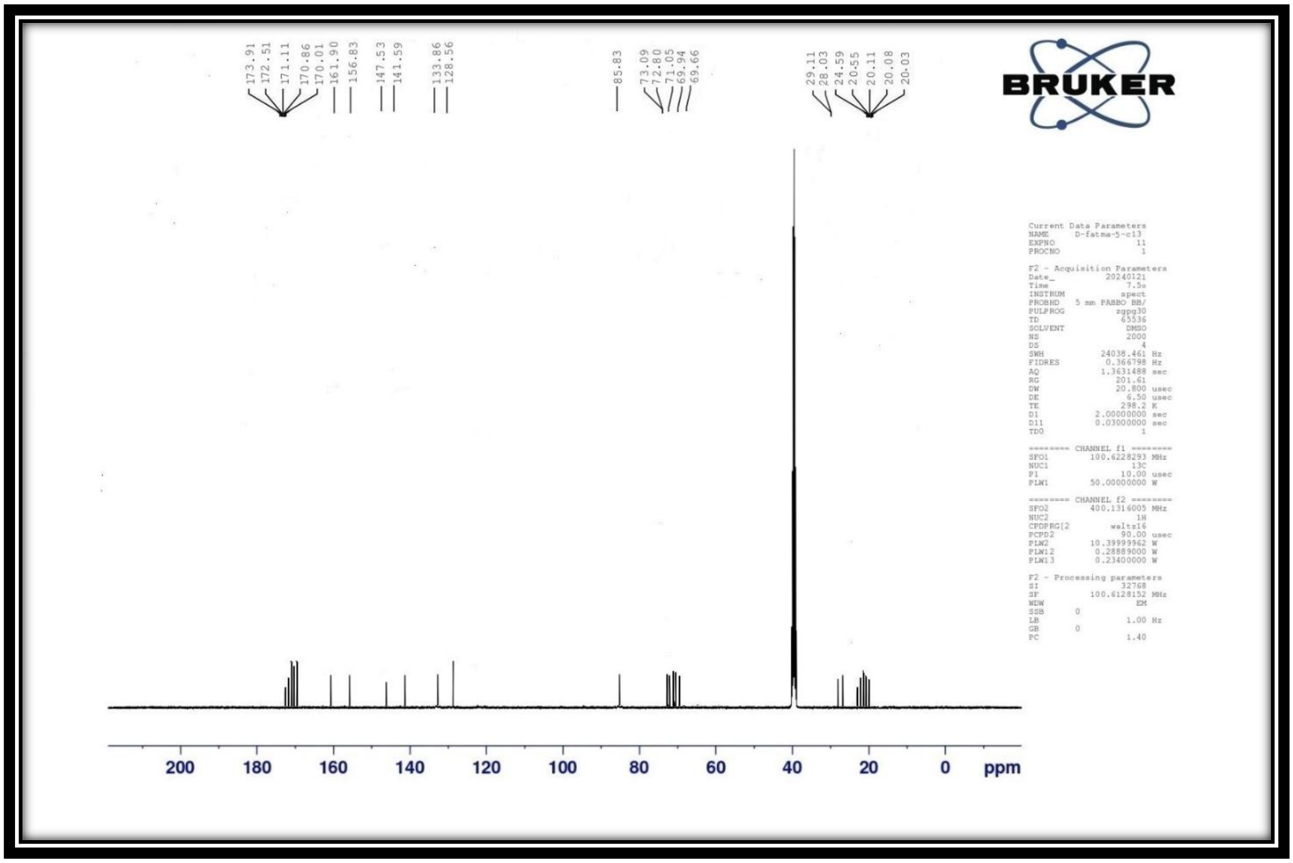

Chart S29: ^{13}C-NMR of Compound 14
